# DegraderTCM: A Computationally Sparing Approach for Predicting Ternary Degradation Complexes

**DOI:** 10.1021/acsmedchemlett.3c00362

**Published:** 2023-12-13

**Authors:** Paolo Rossetti, Giulia Apprato, Giulia Caron, Giuseppe Ermondi, Matteo Rossi Sebastiano

**Affiliations:** University of Torino, Department of Molecular Biotechnology and Health Sciences, CASSMedChem, Piazza Nizza 44, 10126 Torino, Italy

**Keywords:** PROTAC, Ternary complex modeling, Targeted protein degradation, Prediction, Early drug discovery

## Abstract

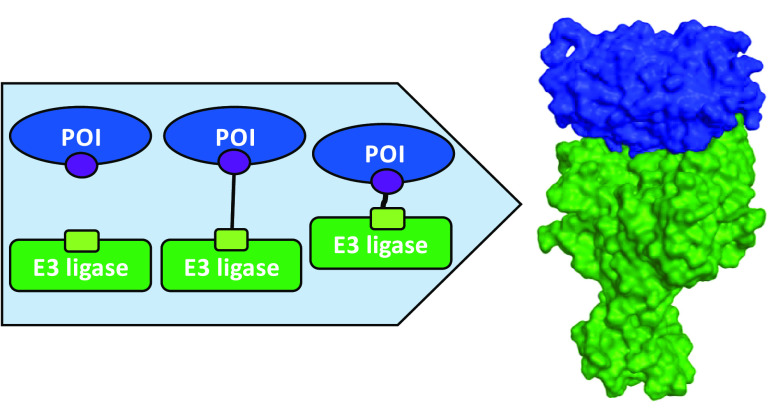

Proteolysis targeting chimeras (PROTACs or degraders) represent a novel therapeutic modality that has raised interest thanks to promising results and currently undergoing clinical testing. PROTACs induce the selective proteasomal degradation of undesired proteins by the formation of ternary complexes (TCs). Having knowledge of the 3D structure of TCs is crucial for the design of PROTAC drugs. Here, we describe DegraderTCM, a new computational method for modeling PROTAC-mediated TCs that requires low computational power and provides sound results in a short time span. We validated DegraderTCM against a selected set of experimentally determined structures and defined a method to predict the PROTAC degradation activity based on the computed TC structure. Finally, we modeled TCs of known degraders holding significance for defining the method’s applicability domain. A retrospective analysis of structure–activity relationships unveiled possibilities for utilizing DegraderTCM in the initial stages of designing novel PROTAC drugs.

Targeted protein degradation through proteolysis targeting chimeras (PROTACs or degraders) represents a novel chemical modality suited to difficult-to-drug targets that has raised interest and advanced to clinics.^1−3^ PROTACs induce the formation of a ternary complex (TC), leading to the ubiquitination of a protein of interest (POI), which is subsequently degraded by the proteasome (Figure 1A).^4^ The PROTACs’ proximity-inducing mechanism of action is allowed by their structure, which is composed of three building blocks:^4^ (1) a POI-binding warhead, often a derivative of known small molecules, (2) an E3 ligand (L^E3^), and (3) a linker connecting the two (Figure 1B).

**Figure 1:**
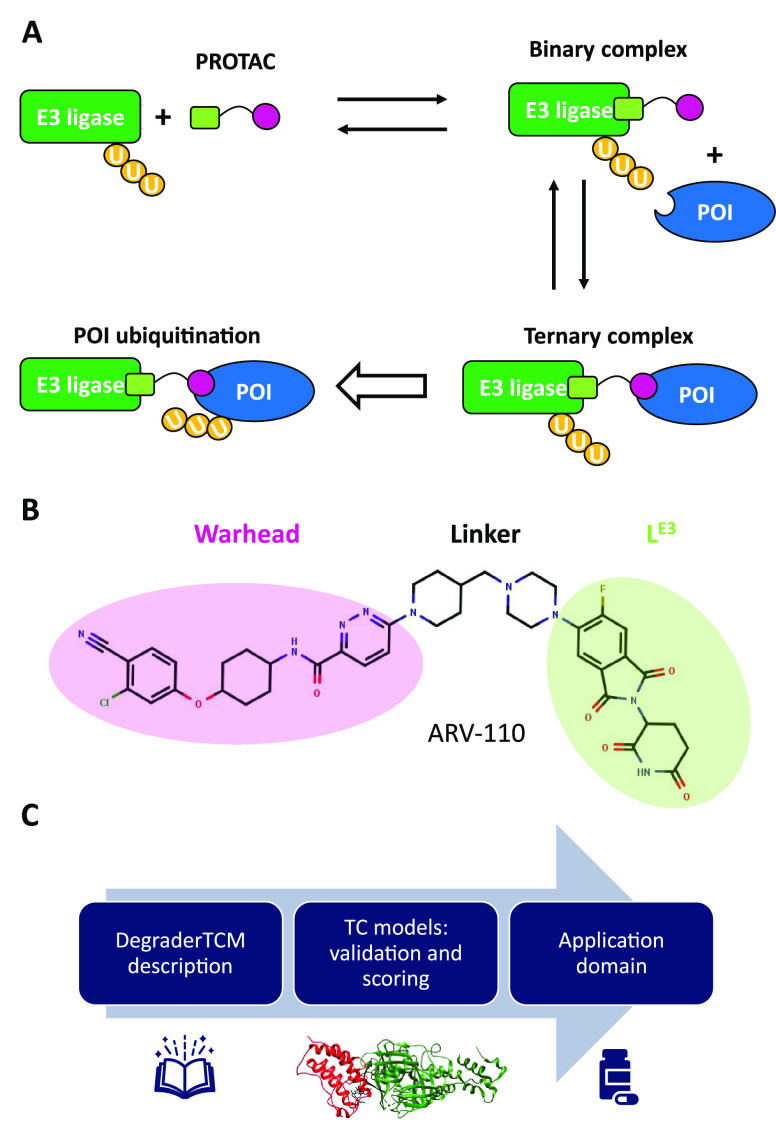
(A) Schematic representation of TC formation and ubiquitination. (B) Structure of the degrader ARV-110, highlighting the PROTAC’s building blocks. (C) Outline of this paper.

PROTACs can be used to induce the degradation of undesirable proteins, thereby allowing, for instance, the ability to counteract the overexpression of oncogenes^5^ or to treat neurodegenerative diseases by degrading misfolded proteins.^6,7^ Moreover, once its function is exerted, each PROTAC molecule can bind other E3 and POI units. This catalytic mechanism shows substoichiometric properties when a single PROTAC molecule induces the degradation of multiple POI units,^7,8^ translating into lower administration doses and less off-side effects. Furthermore, the lower affinity of the warhead is sufficient for promoting degradation in absence of active sites: this allows to potentially target proteins that have previously been considered as undruggable (e.g., transcription factors and scaffolding proteins).^9^

PROTACs are relatively synthetically accessible; however, their design is far from trivial. Not every linker between the warhead and L^E3^ pairs guarantees success, mainly due to the influence of the TC formation, which is a complex and dynamic process where several events take place. For instance, POI and E3 ligases can interact with each other and influence the TC stability. This effect, known as cooperativity, influences the degradation activity by favoring the stability of the complex,^4,10^ even though some limitations have been reported.^11^ Moreover, one must bear in mind that ubiquitination is a complex biological mechanism; that is to say, TC is a necessary, but not sufficient, step for degradation.^12^

Owing to this complexity, there is a high record of inactive PROTACs,^13^ suggesting that determining the structure of the TC is a key aspect for a sound rational design.^10,14^ Until now, few TCs have been crystallized and resolved with X-rays, or at least, only a few experimental structures have been uploaded to the Protein Data Bank (PDB).^15,16^ Moreover, this experimental approach is not suited for early drug discovery purposes, when chemical matter is missing. Thus, there is a stark need for computational strategies in the very early design of new and effective degraders.

Computational methods have already been developed to model TCs.^15−18^ Some of them can be considered “PROTAC-centric” (e.g., methods 1–3 from Drummond and co-workers),^16^ meaning that the conformational properties of the PROTAC drive the construction of the ternary complex, whereas others are “protein-centric”: they use protein–protein docking to drive the TC modeling (e.g., method 4 from Drummond and co-workers).^11^ At present, the best-performing methods result from combinations of the two approaches, such as method 4b^16^ from Drummond and co-workers and PRosettaC.^15^

Although these computational approaches have been used in the design of PROTACs and some of them are included in commercial modeling suites, no one has yet represented a definitive solution for TC modeling.^15,16^ Furthermore, many of the existing methods output several probable TCs models with an appraisable outlook of thorough conformational sampling. This approach is extremely computationally demanding, and in our opinion, this calls for leaner pipelines. Ideally, a method should provide one predicted TC model that, even if not fully exhaustive, captures essential features and is suited for very early drug discovery phases.

To respond to the previously mentioned needs, we here present DegraderTCM, a novel, fast, and easy-to-apply multistep TC modeling method developed in the Molecular Operating Environment (MOE, www.chemcomp.com). DegraderTCM is based on the principle of using the PROTAC linker as a geometric constraint to drive the TC construction. This approach obtained performances comparable to those of other literature methods while still maintaining its simplicity of use. In the next sections, we provide a description of DegraderTCM, validate the method, and show applications of DegraderTCM (as outlined in Figure 1C).

Figure 2 describes the steps of DegraderTCM, and more details are given in the Supporting Information (SI).

**Figure 2:**
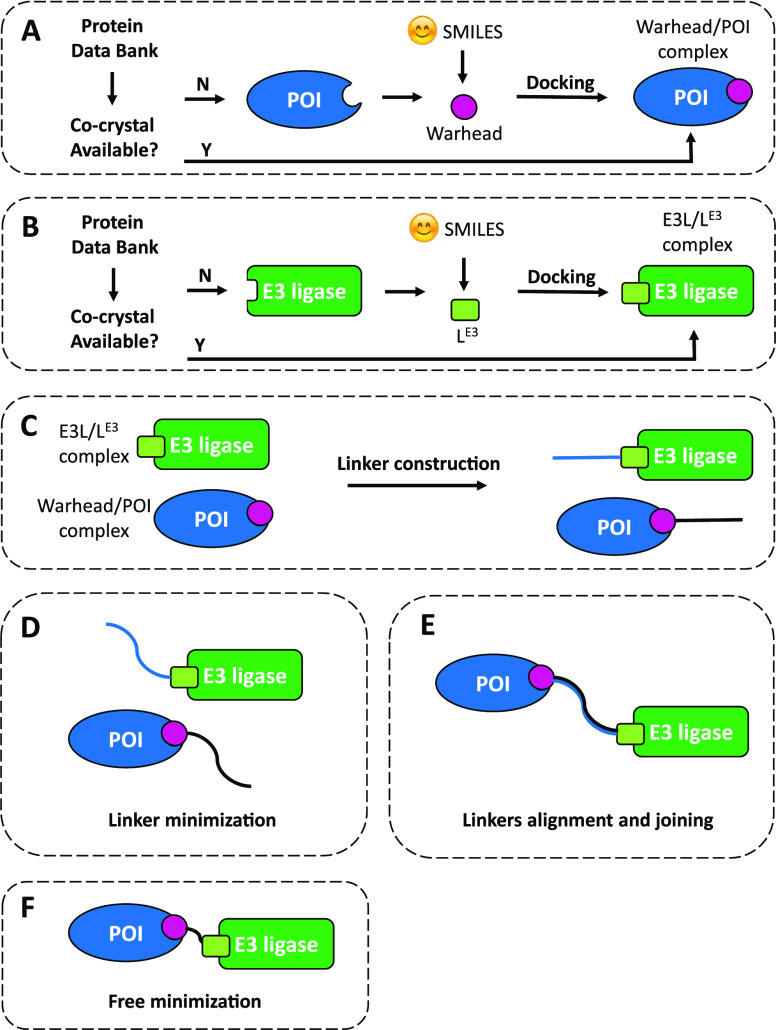
Steps of DegraderTCM. Flowcharts to model the (A) POI–warhead and (B) E3L–L^E3^ complexes. (C) Linker construction of both protein-binding moieties. (D) Separate linker minimization and (E) linker alignment/superposition and joining. (F) Free minimization.

First, the POI–warhead complex was considered (Figure 2A). If a co-crystal structure was available in the PDB, it was used; otherwise, the warhead pose was achieved by a docking procedure implemented in MOE (see the SI). In the second step (Figure 2B), the same procedure as that used for the POI–warhead complex was applied to the E3L–L^E3^ complex.

Then, the linker was built *de novo* upon its full length by employing the MOE builder function and (separately) attaching it to both the warhead and E3L–L^E3^ by respecting the previously identified binding poses (Figure 2C). The POI–warhead–linker and E3L–L^E3^–linker complexes were then separately minimized (Figure 2D). Minimization resulted in linker orientations that reduced clashes and adopted a solvent-facing orientation, which was essential for the subsequent steps.

The two minimized complexes were then merged to form the TC (Figure 2E). The two linkers were superposed using the MOE superpose tool. In short, the reciprocal protein orientation was obtained from the superposition of the linkers. The excess atoms were removed, and the PROTAC structure was connected, obtaining an approximate model of the TC.

In the last step (Figure 2F), a sequence of minimization cycles was employed to refine the model: we started by identifying any eventual protein clashes and applied local minimization rounds to solve them. Then, just the PROTAC molecule was minimized to be accommodated within the proteins. Finally, all atoms in the system were subjected to unrestrained minimization to obtain the final TC model. As a quality control check, the MOE structure preparation tool was used to verify that no residual clashes were present.

The last minimization step often resulted in the reciprocal movement of the two protein structures and the formation of new contacts. In this case, a preliminary visual inspection of the PROTAC was often already informative and could be used to check whether the warhead and L^E3^ maintained optimal binding poses.

To validate our protocol, we first chose five TCs for which a crystallographic structure was present in the PDB. We report the PDB codes, resolutions, PROTAC structures, and POI/E3 pairs of these TCs in Table 1. We chose these TCs because of their use as a validation set for other methods,^16^ the representation of PROTAC chemical diversity (especially in the linkers),^19^ and the different E3 ligases (E3Ls).^20^ The POIs are proteins of great interest: bromodomain-containing protein 4 (BRD4), transcription activator BRG1 (SMARCA4), and Bruton tyrosine kinase (BTK). The E3Ls are the widely recruited Von Hippel-Lindau (VHL), Cereblon (CRBN), and Cellular Inhibitor of Apoptosis (cIAP).

**Table 1:**
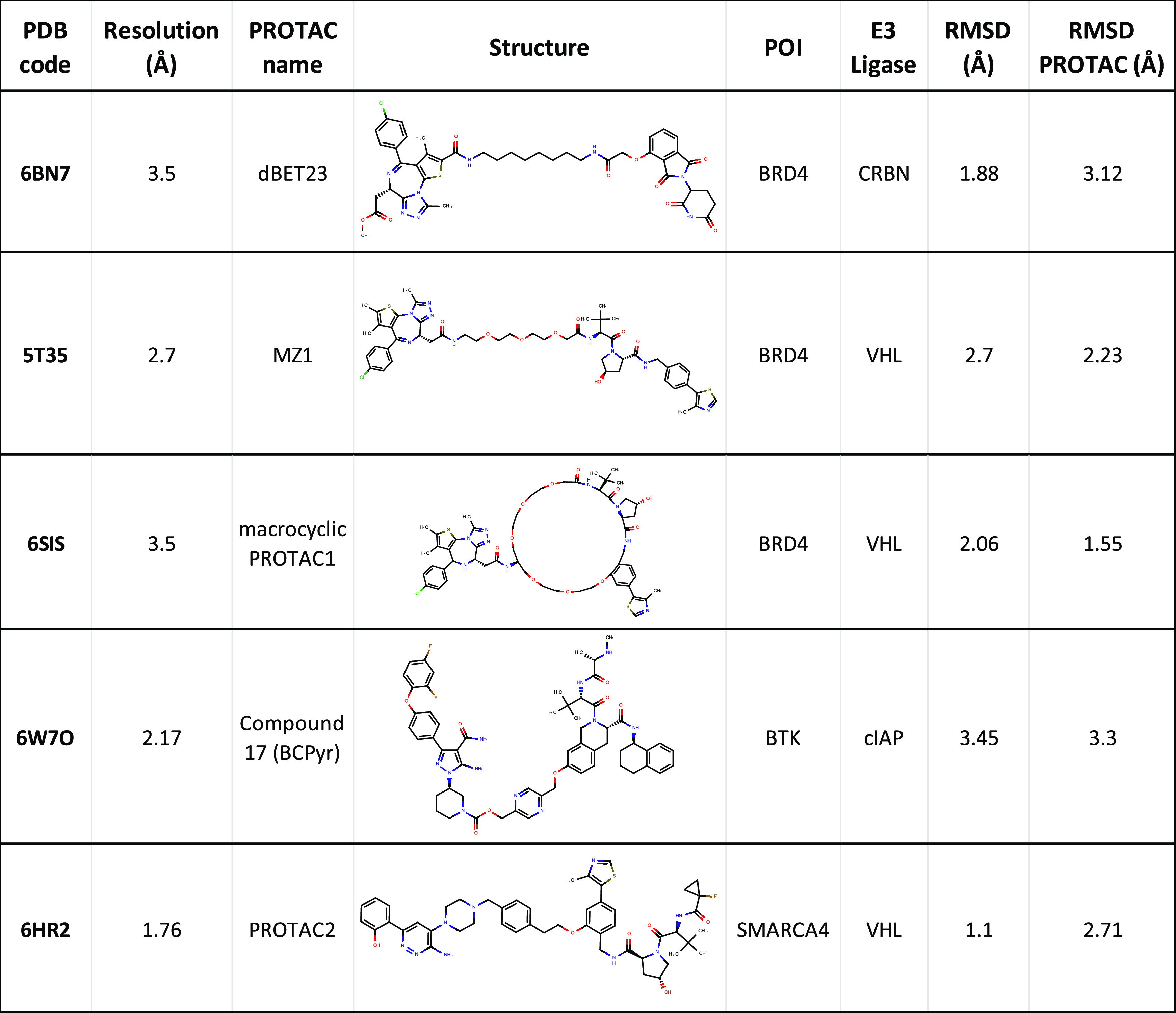
X-ray TC Structures Used to Validate DegraderTCM and Their Resolution and RMSD Values when Superposing Our TCs^a^

a RMSD indicates the whole protein component, while RMSD PROTAC just considers the heavy atoms of the degrader molecule.

Even if they were available in the PDB, the validation of DegraderTCM was carried out by docking the warhead and L^E3^ instead of using the co-crystal structures (see the SI). In this way, we sought to test the robustness of the method in conditions where no previous structural information was available.

The results of DegraderTCM were first evaluated by superimposing the TC models with the corresponding X-ray structures (Figure S2) and calculating the root-mean-square deviation (RMSD) values of both the protein backbone and the PROTAC heavy atoms (Table 1). We obtained protein RMSD values ranging from 1.1 to 3.5 Å (SMARCA4-VHL and BTK-cIAP, respectively). Furthermore, comparable RMSD values were achieved for the PROTAC heavy atoms (Table 1). In general, we can conclude that all values are comparable with the resolution of the X-ray crystal structures and are considerably below the 10 Å threshold established by Drummond and co-workers that discriminates “crystal-like” structures.^16^ In this regard, it is important to underline that DegraderTCM achieved low RMSD values for both VHL- and CRBN-BRD4 complexes, although the existing literature describes lower performances for CRBN TCs^16^ (Table 1).

Then, we compared the interactions individuated by our TC models to those in the crystal structures. Two types of interactions were considered: PROTAC–protein interactions (Figures 3A and S4–S11), and protein–protein interactions (PPIs, Figures 3B and S4–S11). As an example, Figure 3A represents the interaction scheme of the PROTAC dBET23 with BRD4 and CRBN. In this case, it is evident that the modeled TC conserves a large part of the interactions found in the X-ray structure (PDB 6BN7). Similarly, the PPI patterns are comparable by contact surface, type, and number of interactions (Figure 3B), although a detailed analysis of the PPI-involved residues reveals differences (Figure S3A). Similar conclusions can be drawn for the other TC models: small changes occur in the number of hydrogen bonds, salt bridges, and nonbonded contacts (Figure S3B), but the number of involved residues remains comparable in all cases except for PDB 6HR2 (Figure S3C). In our view, such differences arise from small shifts (low RMSD) in the contact surfaces, consistent with part of the pivotal residues remaining (Figure S3–S11). In regard to the TC model involving SMARCA4-VHL and PROTAC2 (Table 1), we that suspect differences arise from poor cooperativity.

**Figure 3:**
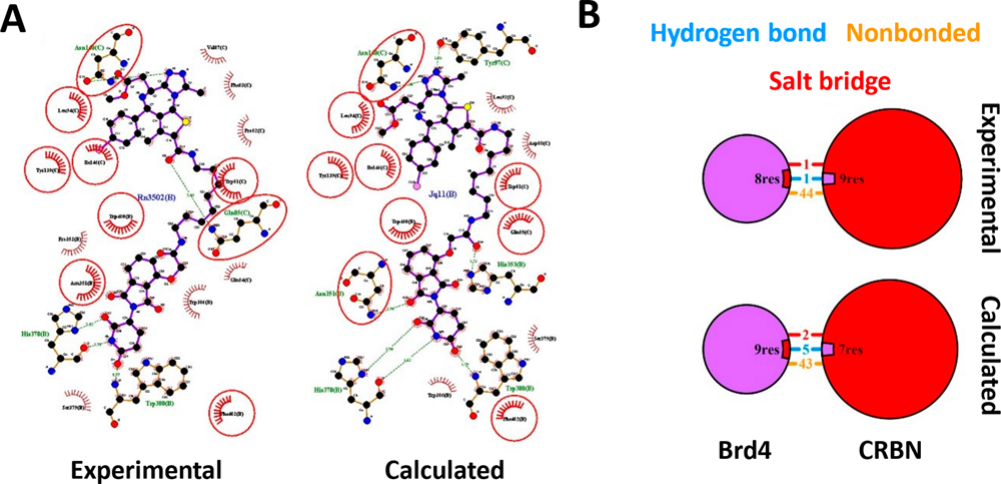
Validation of the BRD4/CRBN TC with dBET23. (A) LigPlot interaction schemes of the PROTAC dBET23 with BRD4 and CRBN comparing PDB 6BN7 and the modeled TC. (B) BRD4 (purple)–CRBN (red) PPI interaction summary from PDB 6BN7 and the modeled TC. The circle size is proportional to the surface area. Contact surfaces are represented by a proportional colored wedge.

Predicting TC structures is particularly helpful in drug discovery for ranking them by degradation efficiency. However, this is not a trivial task for several reasons. First, the propensity to form stable TCs is not the unique factor that determines PROTAC activity.^21^ Second, as a recent analysis of the PROTAC literature discusses, just a few studies effectively measure TC formation when characterizing PROTACs.^22^ Finally, degradation activity data can be obtained with techniques harboring a relevant load of intrinsic variability (e.g., Western blot is affected by cell permeability) or more semiquantitative methods, such as modern cell-based assays.^23^ Thus, degradation data should be regarded as coarse indications.^21,22^ Having said this, we attempted to explain the degradation activity of several literature PROTACs by evaluating the interaction energies with the MOE energy tool (the more negative the energy, the more stable the TC). Details about the specific PROTAC case studies that were used for this purpose are given in Table 2.

**Table 2:** DegraderTCM Scores, Degradation Capacities (Full Data in the SI), Literature Sources, and Peculiarities of Selected Case Studies^a^

TC complex	peculiarity	PROTAC	DegraderTCM TC score	degradation	literature source (PMID)
dardarin/VHL	highly cooperative TC	XL-01126	–142.5	strong	36007011
		XL-01076	–112.57	poor	
		XL-01118	–119.89	poor	
		XL-01149	–119.35	poor	
		XL-01168	–117.8	poor	
ER/VHL	analysis of optimal linker length	ERD-308	–167.65	strong	30990042
		ERD-C18	–188.84	strong	
		ERD-C26	–174.64	strong	
		ERD-C16	–103.82	poor	
		ERD-C17	–111.67	poor	
AR-CRBN	analysis of the optimal exit vector	ARV-110	–169.89	strong	34473519
		ARD-2585	–89.57	strong	
		AR-CRBN-33	–75.33	poor	
AR-VHL	analysis of the overall TC stability	ARD-266	–77.47	strong	31804827
		AR-VHL-1-8	–45.97	poor	30629437

a We considered >50% degradation to define strong degraders.

A score (termed the DegraderTCM score) was defined by accounting for the interactions established by the protein-binding moieties (namely, the warhead and L^E3^) rather than the whole complex. We reasoned that larger approximations are made when considering the whole TC, which would overshadow the key differences among the PROTACs. This is supported by the observation that PPIs can be just partially recapitulated by DegraderTCM (Figure S3). The contributions to the DegraderTCM score are described in eq 1, and details about the calculation are given in the Supporting Information.
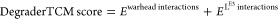
1In eq 1, *E*^warhead interactions^ is the sum of the energy of each interaction established by the warhead in the TC, while *E*^L^E3^ interactions^ is the sum of the energy of each interaction established by the L^E3^.

When considering the POI/E3 pairs in Table 2, for which at least one strong and one poor degrader are present, we can appreciate that the degradation efficiency is recapitulated by the DegraderTCM score (Figure 4). This is particularly relevant, as it applies to different degrader series.

**Figure 4:**
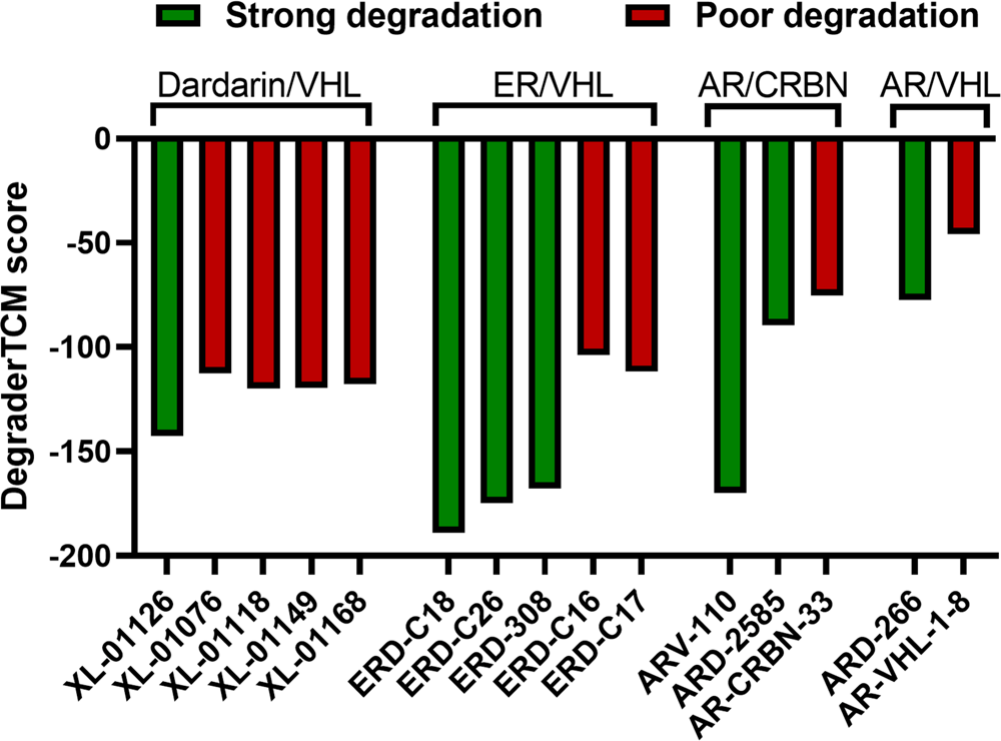
DegraderTCM score (kcal/mol) for 15 PROTACs for which TCs were modeled.

With the definition of the DegraderTCM score, we provided a step toward ranking active and inactive PROTACs (Figure 4). This reinforces the idea that degraders with binding poses that preserve key native interactions of the warhead and L^E3^ can form stable TCs and suggests that this is sufficient for predicting their degradation activity.^24^

Next, we report more details about the four POI/E3 pair groups in Table 2 to address specific questions about the investigated systems.

First, we wanted to test the performance of DegraderTCM with highly cooperative complexes, so we modeled TCs of five PROTACs targeting dardarin (Table 2 and Figure 5A), a protein involved in Parkinson’s disease,^25^ and recruiting VHL. The considered degraders have been recently developed by Liu and co-workers,^26^ who individuated XL-01126 as the most potent derivative of their series. As negative controls, we considered XL-1168 and XL-1076, characterized by short and rigid linkers, and XL-1118 and XL-1149, which have longer and more flexible linkers (Figure S1). The peculiar protein–protein interface of the dardarin/VHL TCs shows numerous PPI contacts (Figures S12–S21) involving two different surfaces of VHL with dardarin wrapping around the E3L (e.g., XL-01126 in Figure 5A). For such highly cooperative complexes, even if strong differences in degradation are present (Table S2), more similar DegraderTCM scores were found. However, the lowest value of the series belongs to XL-01126 (−142.5 kcal/mol), which was the most active compound.

**Figure 5:**
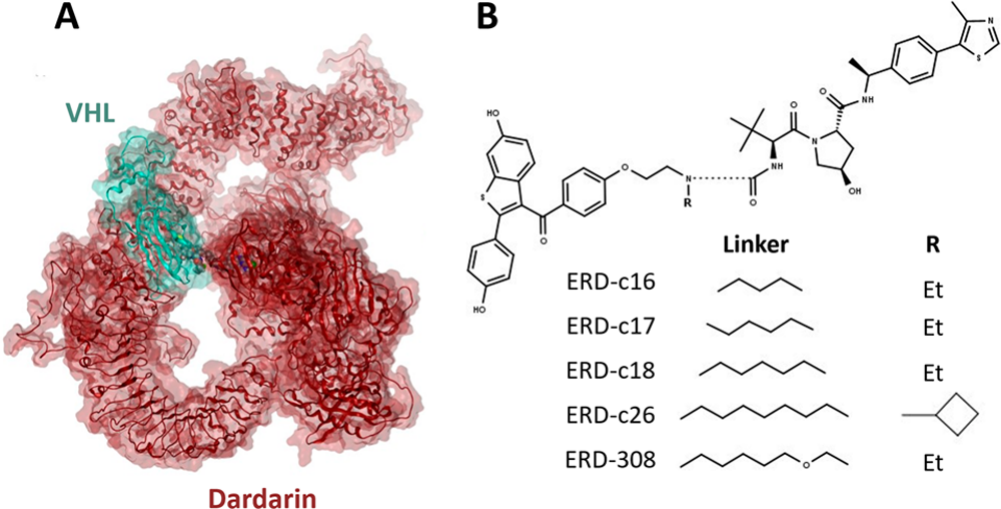
(A) Ternary complex of XL-01126, involving dardarin (red) and VHL (cyan). (B) Structure of ER/VHL degraders, showing the exploration of different linker lengths.

One of the most important steps in PROTAC design is the determination of the linker characteristics to promote degradation.^19^ To address this issue with DegraderTCM, we modeled TCs from a series of ER/VHL degraders (Table 2 and Figure S1). The most active compounds were ERD-308, ERD-C18, and ERD-C26.^27^ During the development of the series, some of the sources of chemical diversity were the progressively increasing linker length and flexibility, which were achieved by the addition of carbon units (compounds ERD-C16, ERD-C17, and ERD-C18; see Figure 5B). In this case, the DegraderTCM score suggests that ERD-C18 (5 carbon atoms linker) forms the most stable TC (Table 2). By examining the specific interactions established by the PROTACs in the TCs, it can be determined that shorter linker lengths break key interactions of the warhead (Figures S22–26). To strengthen our point, we report TCs for two additional compounds representing positive controls derived from linker expansions: ERD-308 and ERD-C26 (Figure S1).^27^ In ERD-308, an oxygen atom was included in the linker, while in ERD-C26, the linker had a cyclobutyl moiety (Figure 5B). In these cases, the score and specific interactions (Figure S22–S26) agree with the degradation data (Table 2), supporting that our TC models could be potentially employed to expand compound libraries.

TC models can also provide important insights into the optimal exit vector (EV). The EV is commonly referred to as the direction assumed by the linker when the warhead sits in the optimal binding pose. For this reason, we modeled TCs of three PROTACs targeting the androgen receptors (ARs) ARV-110, ARD-2585, and AR-CRBN-33 (Table 2 and Figure 6A). The selected PROTACs share the same L^E3^ to recruit CRBN, have similar warheads, and rigid linkers. However, ARV-110 and ARD-2585 are strong degraders (ARV-110 is in clinical trials), while the degradation of AR-CRBN-33 is poor (Table 2).^28^

**Figure 6:**
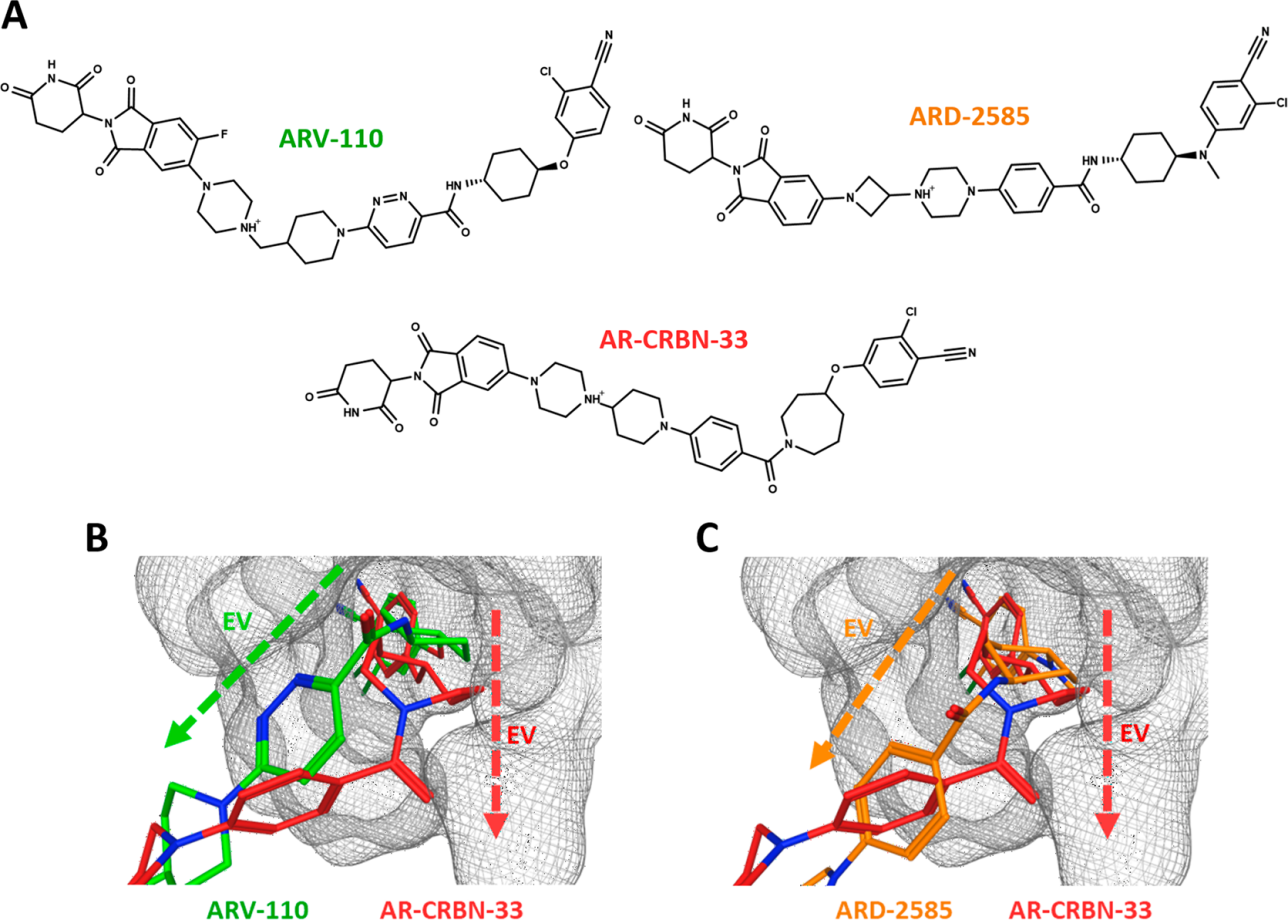
(A) Structures of ARV-110, ARD-2585, and AR-CRBN-33. (B) Comparison between ARV-110 (green) and AR-CRBN-33 (red). (C) Comparison between ARD-2585 (orange) and AR-CRBN-33. The gray mesh defines the warhead binding pocket on the AR, and the dashed arrows define the Exit Vectors (EV).

A close analysis of the AR pocket surface (Figure 6B,C, gray mesh) and the relative position of the PROTAC linkers reveals a completely different exit vector of AR-CRBN- 33 compared to that of ARV-110 (Figure 6B) and ARD-2585 (Figure 6C). This is likely due to the steric hindrance of the azepane ring and the consequent conformational effect. Our observations agree with the degradation data, and they are reflected in the TC energy scoring (Table 2).

VHL-recruiting PROTACs have been developed too.^5,29^ Here, we briefly report the comparison between ARD-266 and AR-VHL-1-8 (Figure S1), a strong and poor degrader, respectively (Table 2).^5^ This case is emblematic of situations where TC models are helpful for the comparison of less structurally related PROTACs, such as ARD-266 and AR-VHL-1-8. In similar cases, it is difficult to conclude much by observing specific interactions (see Figures S33–S36). However, the DegraderTCM score provides the correct stability ranking, which is in agreement with the degradation capacity (Table 2).

The previous examples highlight the power of DegraderTCM for rationalizing the degradation activity through specific aspects of TC formation. We now want to answer the question of whether these considerations can be generalized. To test the application domain, we considered the PROTAC-DB database, containing information for more than 3000 PROTACs.^30^ We reasoned that a systematic analysis would ensure coverage of the present “protaccable” proteome,^9^ highlighting the application domain of DegraderTCM (details are given in the SI, Methods section).

Following a previous investigation, we privileged DC50 data,^22^ obtaining 905 PROTACs and 38 POI classes by function (Figure S39). Selection was based on the presence of active and inactive compounds (Figure S40) and yielded 12 PROTAC pairs, which are representative of the POIs in Figure 7A. Together with the systems discussed above, we reached a coverage of 16 target classes. As a remark, the chemical diversity of the chosen degraders was also in line with the PROTAC-DB content, as highlighted in Figure 7B, reporting the chemical space from seven representative molecular descriptors.^13^

**Figure 7:**
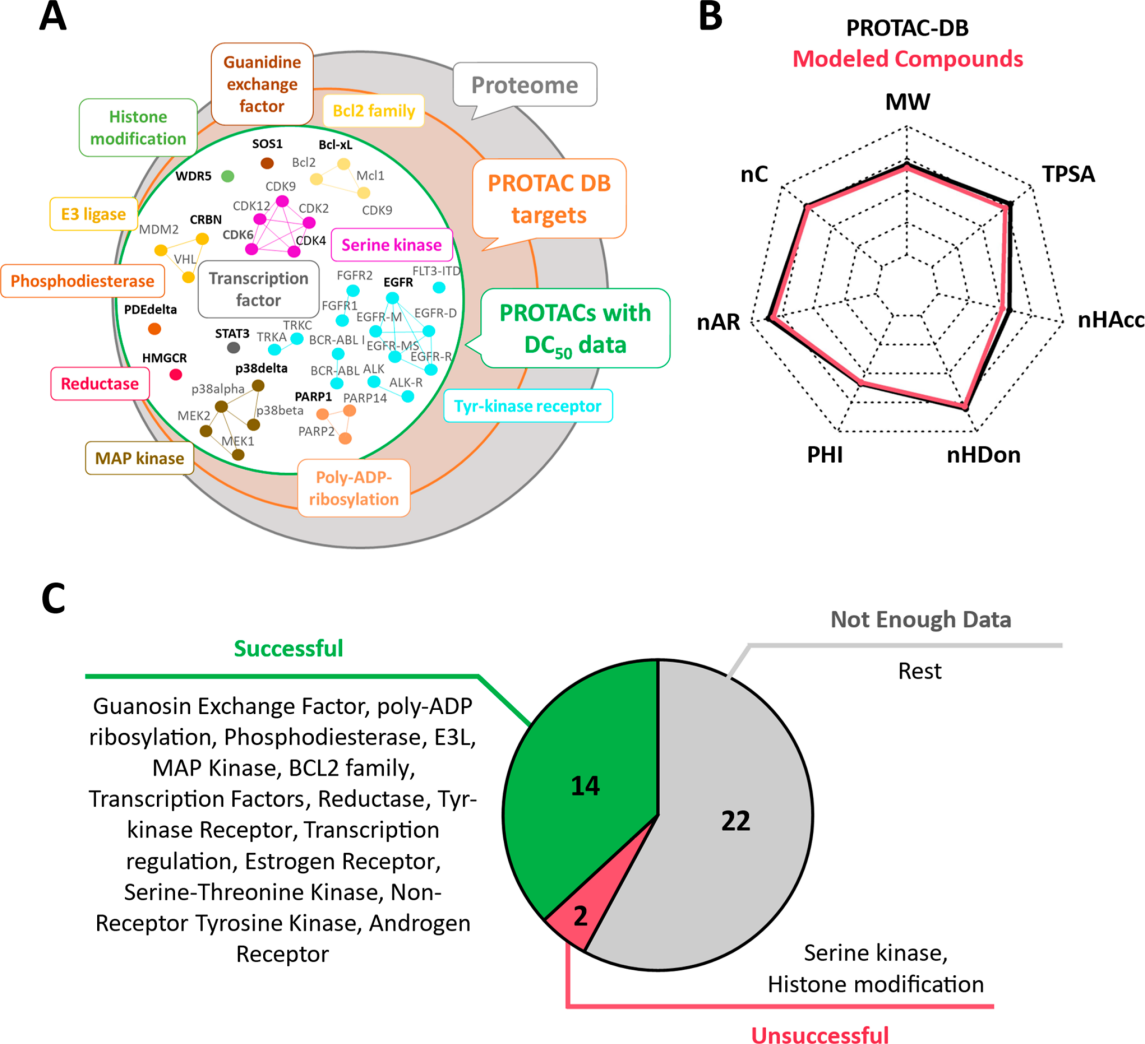
Application domain. (A) Venn diagram reporting selected representative targets to test the application domain. In bold are the selected POIs. (B) Chemical diversity of the tested PROTACs. (C) Rationalization domain based on the previously modeled TCs and the PROTAC-DB selection in (A).

The DegraderTCM scores could, in large part, explain the degradation differences (Table S3) and show a trend of inverse correlation with the DC50 difference (data not shown). Altogether, the degradation activity of 14 of the 16 degrader pairs was explained, representing 87.5% of the tested “protaccable” protein space (Figure 7C). Regarding the two mispredicted pairs, we interpret them as follows: WDR5 (a histone modifier) is part of large protein complexes and may undergo huge conformational changes, challenging the minimization procedure. The serine kinase CDK6 displays highly conserved binding sites, and the readout could potentially suffer from selectivity issues.

By presenting DegraderTCM, we have shown that crystal-like quality TC models, reproducing experimental data, can be obtained in a relatively simple way. Furthermore, by selecting relevant examples, we validated the use of such models and provided a scoring method to interpret them. In this section, we briefly frame DegraderTCM in the landscape of the existing methods and suggest how to interpret the models and potential uses in drug discovery.

Undoubtedly, DegraderTCM can be described as “PROTAC-centric”, as it is based on the capacity of the linker to accommodate the whole PROTAC structure and respect the native binding poses of the warhead and L^E3^. A logical consequence is that the best performance is achieved for rigid linkers due to the restricted conformational space. However, this issue (also reported for other TC modeling methods)^15,16^ seems to just moderately affect the models and the extracted information content, as the validation against X-ray structures and the analysis of ER/VHL series show. We interpret this as an effect of the minimization cycles, still allowing us to model reasonable PPIs by finding local minima, as the case of the highly cooperative dardarin shows. Of course, we are aware that DegraderTCM may overlook huge protein conformational changes and struggle to model PPIs in less cooperative TCs. This limitation is common for methods involving rigid-body protein docking but not for molecular dynamics-based protocols: in such cases, we advise one to budget larger computational resources.^31^

Furthermore, we showed that, even if sometime approximative, the DegraderTCM score, an energy estimation of the protein-binding moieties, can rationalize known degradation activity within PROTAC series, or at least distinguish active/inactive pairs. When no reference pairs are available, one should investigate specific interactions established by the PROTAC in the TC model and compare them with X-ray structures of the protein-binding moieties for qualitative conclusions. We hypothesize that similar comparisons would be useful in terms of binding energy, leading to quantitative considerations (see Figure S41 for more details). This aspect will be the subject of further investigation in the future. We believe that this approach is particularly suited for very early drug discovery phases.

The final question is how and when to use DegraderTCM. The POI space within the investigated proteome seems sufficiently wide for guaranteeing good coverage of multiple targets. By nature, the method is designed to require common superposition and minimization algorithms and low computational power (even a personal laptop can be employed) while still providing acceptable TC models in a short time. As a final consideration, we designed (and tested) DegraderTCM in MOE, starting from structures in the Protein Data Bank so that a single software suite could be employed. However, we cannot exclude that analogue pipelines could work with other (free) software pieces and with AlphaFold structures. Overall, DegraderTCM is suggested to be used for driving the expansion of existing PROTAC series (e.g., to optimize the linker length) or when the first compounds are to be designed and initial decisions must be taken (e.g., optimal exit vectors). This means that DegraderTCM is particularly suited for very early drug design when little prior information is available.

## Supporting Information Available

Formula strings of the studied compounds (XLSX)
Data for the TC models created in this study (ZIP)
Materials, protocols, supplementary figures, and supplementary tables (PDF)

## Abbreviations

AR: androgen receptor
CRBN: Cereblon E3 ligase
E3L: E3 ligase
EV: exit vector
GEF: guanidine exchange factor
HB: hydrogen bond
L^E3^: ligand E3
PDB ID: Protein Data Bank ID
POI: protein of interest
PROTAC: proteolysis targeting chimera
TC: ternary complex
TPD: targeted protein degradation
VHL: Von Hippel-Lindau E3 ligase
