# Supplementary material for: DegraderTCM: A Computationally Sparing Approach for Predicting Ternary Degradation Complexes
Source: ACS Med Chem Lett. 2023 Dec 13;15(1):45–53. doi: 10.1021/acsmedchemlett.3c00362 (PMC10788944; doi:10.1021/acsmedchemlett.3c00362)
Supplement: Supplementary file 3 — ml3c00362_si_003.pdf [file ml3c00362_si_003.pdf]

# Supporting information

# DegraderTCM: a computational-sparing approach for predicting Ternary Degradation Complexes

*Paolo Rossetti, Giulia Apprato, Giulia Caron, Giuseppe Ermondi and Matteo Rossi Sebastiano**

University of Torino, Molecular Biotechnology and Health Sciences Dept., CASSMedChem, piazza Nizza 44, 10126 Torino, Italy.

*Corresponding author, Dr. Matteo Rossi Sebastiano (matteo.rossisebastiano@unito.it)

## List of contents

### Supplementary Files

SMILES.csv, contains formula strings.

### Materials and Protocols

Advanced protocols and Methods description

### Supplementary Figures

Figure S1: modelled PROTAC structures

Figure S2: superposition of our models with crystal structures

**Figure S3.** Protein-protein interactions of the TC models employed for the validation.

Figure S4: PROTAC interactions and protein contact surface for MZ1/Brd4/VHL (PDB 5T35)

Figure S5: PPIs in MZ1/Brd4/VHL (PDB 5T35)

Figure S6: PROTAC interactions and protein contact surface for cPROTAC1/Brd4/VHL (6SIS)

Figure S7: PPIs in cMZ1/Brd4/VHL (6SIS)

Figure S8: PROTAC interactions and protein contact surface for PROTAC2/SMARCA4/VHL (6HR2)

Figure S9: PPIs in PROTAC2/SMARCA4/VHL (6HR2)

Figure S10: PROTAC interactions and protein contact surface for compound-17/BTK/cIAP (6W7O)

Figure S11: PPIs in compound-17/BTK/cIAP (6W7O)

Figure S12: PROTAC interactions and protein contact surface for the TC of XL-01126

Figure S13: PPIs in the TC of XL-01126

Figure S14: PROTAC interactions and protein contact surface for the TC of XL-01168

Figure S15: PPIs in the TC of XL-01168

Figure S16: PROTAC interactions and protein contact surface for the TC of XL-01149

Figure S17: PPIs in the TC of XL-01149

Figure S18: PROTAC interactions and protein contact surface for the TC of XL-01076

Figure S19: PPIs in the TC of XL-01176

Figure S20: PROTAC interactions and protein contact surface for the TC of XL-01118

Figure S21: PPIs in the TC of XL-01118

Figure S22: PROTAC interactions and protein contact surface for the TC of ERD-308

Figure S23: PPIs in the TC of ERD-308

Figure S24: PROTAC interactions and protein contact surface for the TC of ERD-c17

Figure S25: PPIs in the TC of ERD-c17

Figure S26: PROTAC interactions for ERD-c16 and ERD-c18

Figure S27: PROTAC interactions and protein contact surface for the TC of ARV-110

Figure S28: PPIs in the TC of ARV-110

Figure S29: PROTAC interactions and protein contact surface for the TC of ARD-2585

Figure S30: PPIs in the TC of ARD-2585

Figure S31: PROTAC interactions and protein contact surface for the TC of AR-CRBN-33

Figure S32: PPIs in the TC of AR-CRBN-33

Figure S33: PROTAC interactions and protein contact surface for the TC of ARD-266

Figure S34: PPIs in the TC of ARD-266

Figure S35: PROTAC interactions and protein contact surface for the TC of AR-VHL-1-8

Figure S36: PPIs in the TC of AR-VHL-1-8

Figure S37: PROTAC interactions and protein contact surface for the TC of ARV-471

Figure S38: PPIs in the TC of ARV-471

Figure S39: Targets in the classes with DC50 data

Figure S40: PROTAC-DB target classes with DC50 data

Figure S41: Conserved interactions in the TC model of ARV-471

**Supplementary Tables**

Table S1: PDB codes of reference structures.

Table S2: literature degradation data.

Table S3: Additional PROTACs.

## Materials and Protocols

### *Docking protocol*

The target protein was prepared with the Structure preparation tool of MOE, hydrogen atoms are added to the structures and the protonation state adjusted at pH 7.4 (Protonate 3D module). The ligand was prepared in MOE by assigning charges and protonation, and the structure was minimized with MMFF94x (RMS gradient 0.1 kcal/mol/ $Å^2$).

The docking engine in MOE allows to determine the 3D coordinates of a molecule in the binding pocket (the pose) in two steps: poses generation and refinement. Poses generation was made using the Triangle matcher algorithm, generating 30 poses, the timeout time was set as default. The scoring for the pose generation step was set as London dG. The refinement was performed using an induced fit for all the docking experiments, the pocket coordinates correspond to a 6x6x6Å box in which just side chains were allowed to move under tethering constraint. The refinement takes the 30 poses generated by the previous step and after calculating the energy score (defined as GBVI/WWSA) retains the best 5 poses. The best-pose

was selected as the one with the lowest S score, and this was considered as the ligand binding pose for starting ternary complex modelling.

To get crystal-like poses of the Brd4 warhead we performed a pharmacophore-biased docking of JQ1 in the two Bromodomains of Brd4. The structure of JQ1 in the first bromodomain was retrieved from PDB ID 3MXF, the second from PDB ID 3ONI.

A common pharmacophore file (.ph) was created (tool: Pharmacophore/Query editor, parameters: one hydrophobic/aromatic feature (clorophenyl ring), a first HB-acceptor feature (N2 in the triazole ring), a second HB-acceptor feature (diazepin N). The target structure was prepared and protonated at pH 7.4. The ligand was protonated and minimized (MMFF94x RMS gradient 0.1 kcal/mol/ Å$^2$) and finally included in a separated .moe file. The pocket was defined as “Ligand atoms” allowing to restrict the docking to JQ1 pocket. The pharmacophore was inserted as .ph file in MOE. The chosen placement method was Triangle matcher generating 30 poses and the refinement with Induced Fit generated 5 poses. The output was included in a MOE database file .mbd and the best pose was the one with the lowest S score.

*Editing of the PROTAC structure*

The two linker moieties were constructed with the MOE builder function. After separate minimization, they were aligned with the structure superpose panel by choosing three non-

adjacent linker atoms. The MOE molecule Builder was used to erase the duplicated atoms of the linker and to create a new bond, joining the two PROTAC parts. Bond length was adjusted with a minimization cycle.

*Minimization protocols*

The application of a minimization protocol to a specific subset of atoms was performed by selecting the chains/atoms subset and by selecting in the minimization panel of MOE vv2022 (www.chemcomp.com) the option “selected”. All operations were carried with the MMFF94x forcefield, as implemented in MOE. Prior to any minimization, hydrogen atoms were added to the structures and the protonation state was adjusted at pH 7.4 with the MOE tool “Protonate3D”.

Local portions of the proteins were minimized in the built-in function (Protein Minimize) as implemented in MOE vv 2022 to resolve local clashes. Those were identified by using the MOE tool called Protein Preparation and the atom selection was extended to +/- 4 residues and 4.5 Å around them.

PROTAC structures and whole complexes were minimized with the tool “Energy minimize” by employing a steepest descent algorithm with an RMS gradient of 0.05 kcal/mol/ $Å^2$ and by

treating planar systems as rigid bodies. The rest of the settings were standard. No tethering was applied.

*Visual inspection, clashes search, and PPI visualization*

All TCs were visually inspected using MOE. MOE Superpose tools was used to superpose the two binary complexes during the TC modelling. Possible clashes with the MOE structure preparation tool were looked for. PROTAC-protein interactions were visualized with LigPlots, while we employed PDBSum (https://www.ebi.ac.uk/thornton-srv/databases/pdbsum/Generate.html) reports for PPIs.

*Scoring with Hooman's interact 7.0*

The scoring of the PROTACs-proteins interactions was made using the svl script named "Interaction Forces and Energies" found at https://svl.chemcomp.com. The program calculates and displays the atomic interaction forces between the ligand and the pocket, as well as the per residue interaction energies of such interactions.

It was used, on the minimized complexes, with default parameters, except for the site radius (Site R) that was set to 4.5 Å. The script was run for separate portions of the PROTAC, allowing to retrieve different scores for the warhead, for the linker and for the $L^{E3}$. We have evaluated

the affinity of the warhead for the POI, of the linker for both POI and E3, and of the $L^{E3\ for}$ the E3L.

**Selection within the PROTAC-DB for application domain testing**

The PROTAC-DB database was downloaded from http://cadd.zju.edu.cn/PROTACdb/about on 12/10/2023. All PROTACs not reporting DC50 data were discarded. The remaining ones were classified according to the target protein into 38 different classes; the GeneCards database (https://www.genecards.org/) was used as a reference for classification purposes. A PROTAC PAIR was considered as strong/poor degrader representative of each class. All classes including only one compound were discarded and are not considered.

The PROTAC pairs were selected according to the following criteria:

- the difference in activity (Δ-DC50) between the two PROTACs should be as high as possible
- both PROTACs need to share the same target
- both PROTACs need to share the same E3 ligase recruited

Pairs having low Δ-DC50 were discarded. For each pair a crystal pose of the warhead or a small molecule highly resembling the warhead was searched from the Protein Data Bank (PDB,

https://www.rcsb.org/). PROTACs not having a warhead present in the PDB as a crystal pose were discarded and were selected again considering the three criteria.

### Safety statement

No unexpected or unusually high safety hazards were encountered during this work.

## Figures

**Figure S1.** Structure formula of the PROTACs for which TCs were modelled.

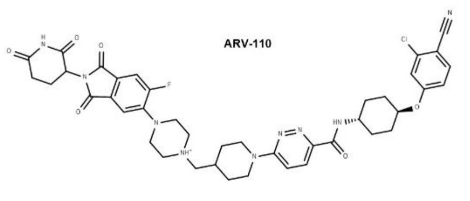

**ARV-110**

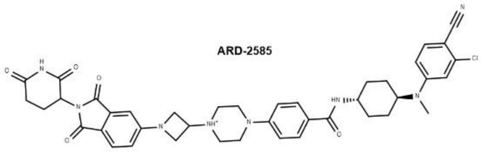

**ARD-2585**

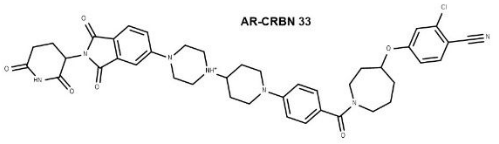

**AR-CRBN 33**

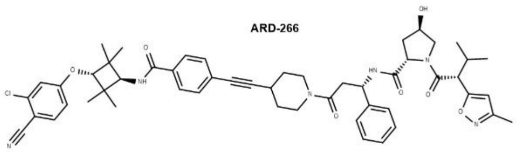

**ARD-266**

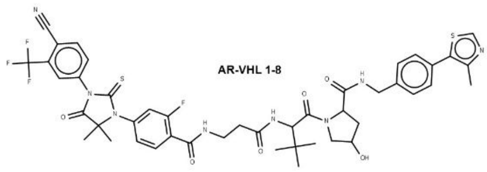

**AR-VHL 1-8**

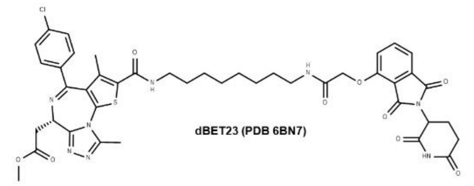

**dBET23 (PDB 6BN7)**

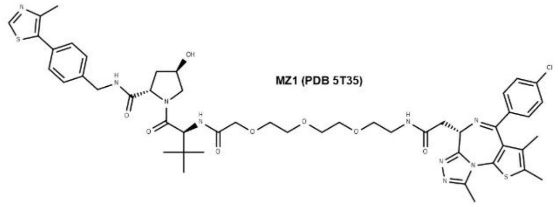

**MZ1 (PDB 5T35)**

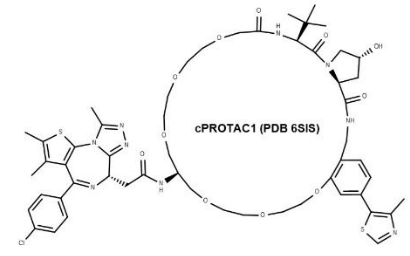

**cPROTAC1 (PDB 6SIS)**

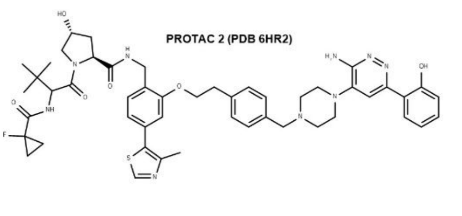

**PROTAC 2 (PDB 6HR2)**

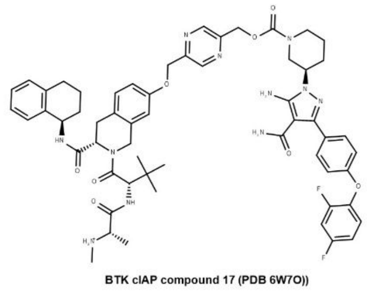

**BTK cIAP compound 17 (PDB 6W7O))**

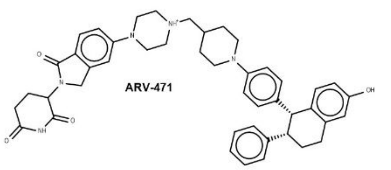

**ARV-471**

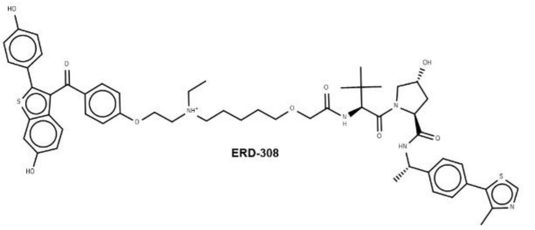

**ERD-308**

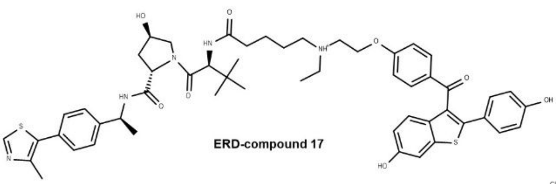

**ERD-compound 17**

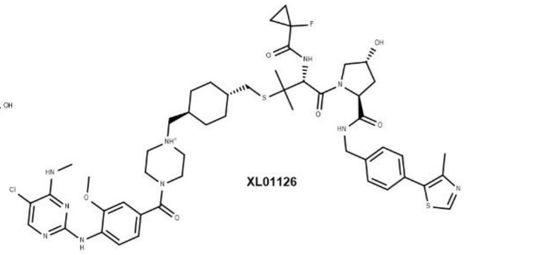

**XL01126**

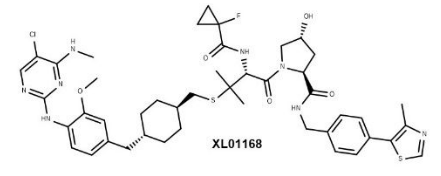

**XL01168**

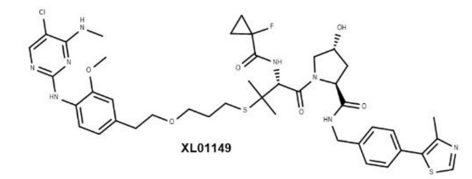

**XL01149**

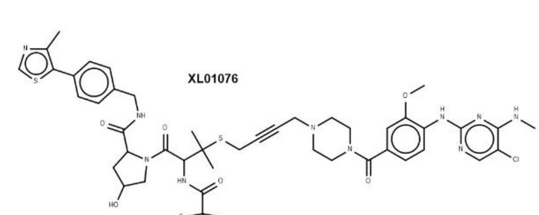

**XL01076**

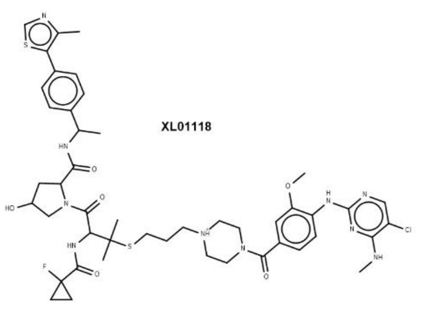

**XL01118**

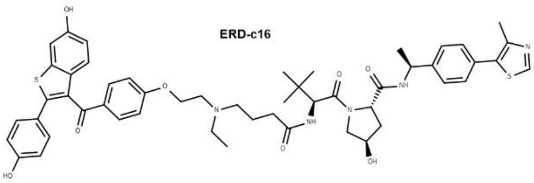

**ERD-c16**

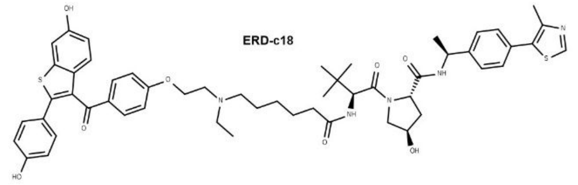

**ERD-c18**

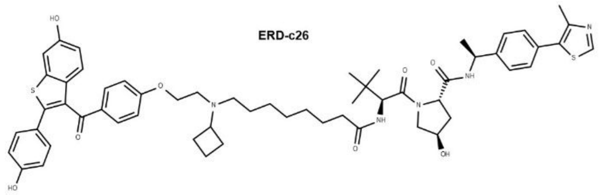

**ERD-c26**

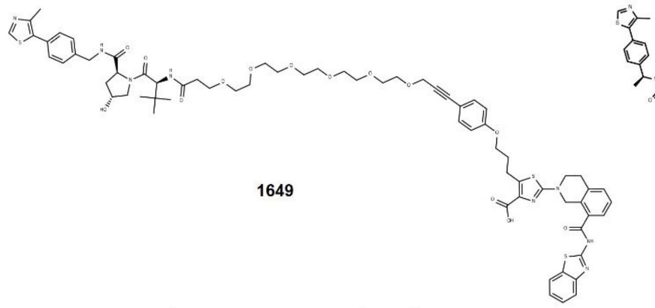

1649

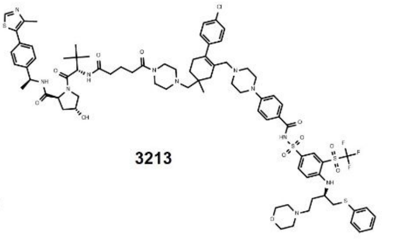

3213

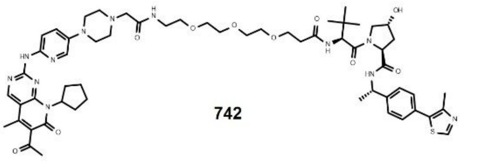

742

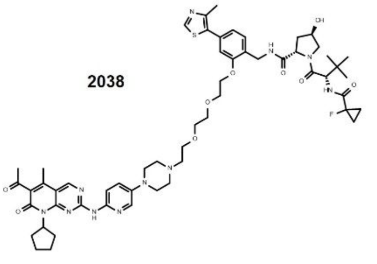

2038

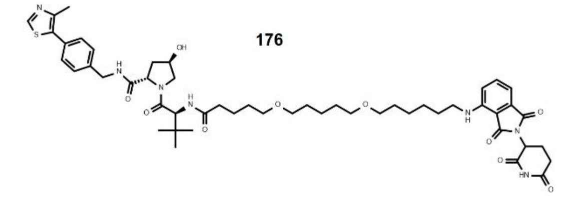

176

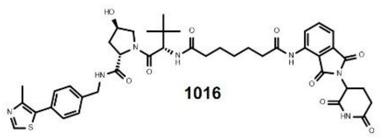

1016

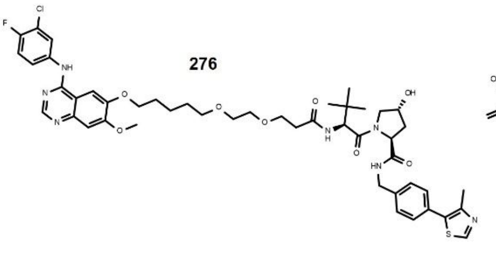

276

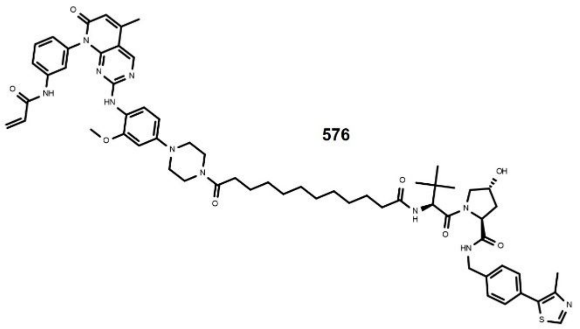

576

1108

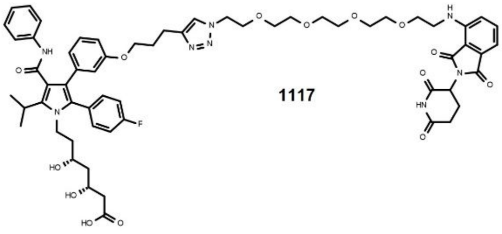

1117

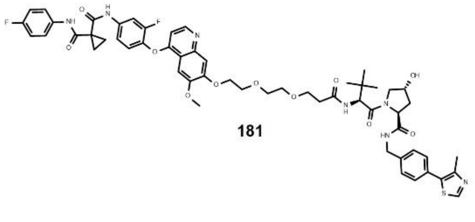

181

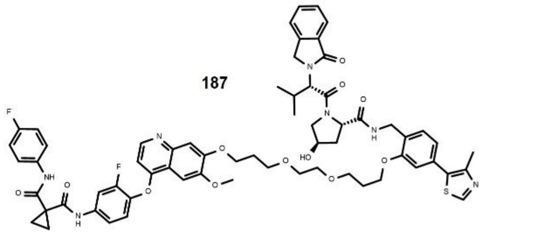

187

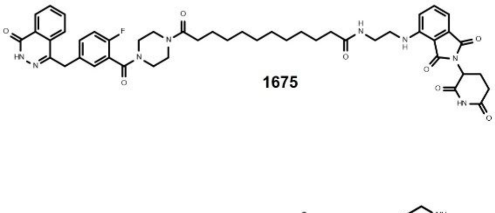

1675

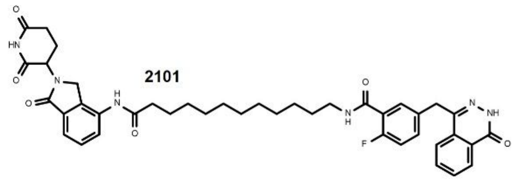

2101

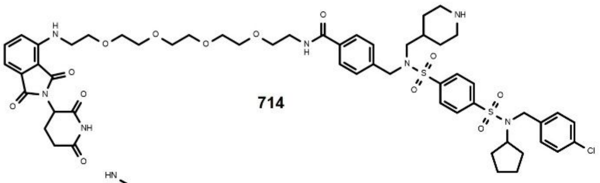

714

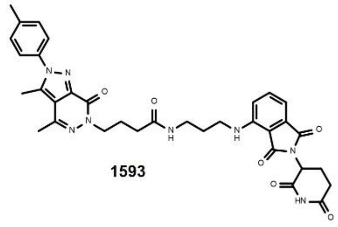

1593

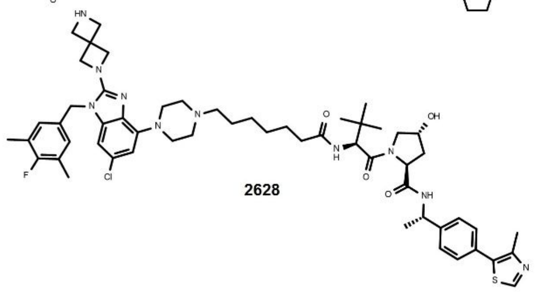

2628

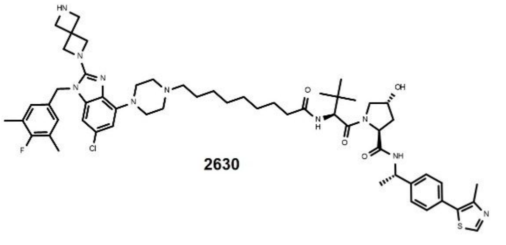

2630

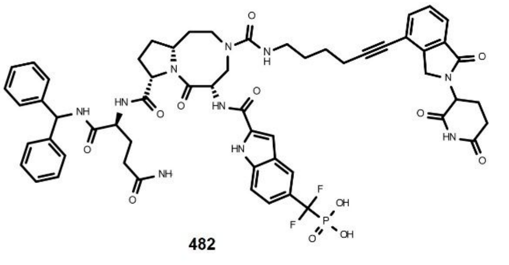

482

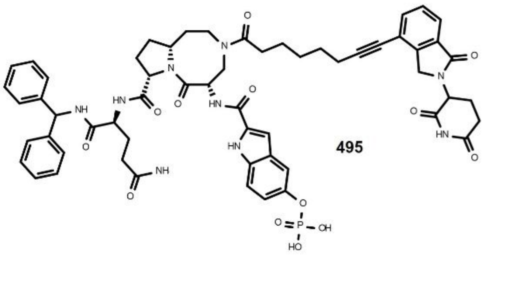

495

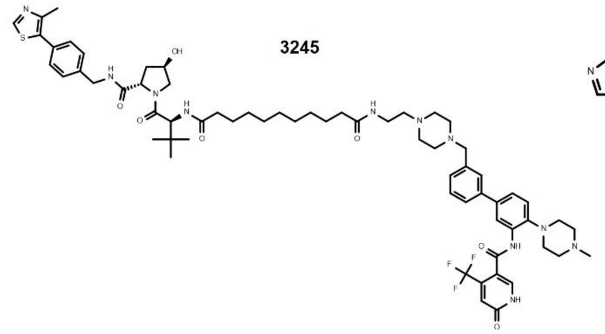

N
S
H
N
O
OH
N
O
H
N
O
O
H
N
N
N
N
NH
O
F
F
F
N
NH
O
N


**3245**

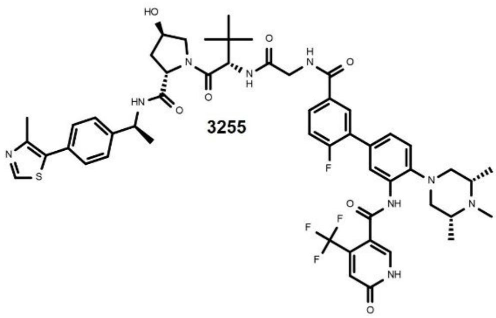

HO
N
O
H
N
O
H
N
O
HN
O
N
S
F
N
NH
O
F
F
F
NH
O
N


**3255**

**Figure S2.** Superposition between crystal structures of TCs (yellow) and the respective models (magenta) visually highlighting the imprecisions of modeling. N.B. The pose for Brd4 and VHL encompasses both MZ1 and macrocyclic MZ1.

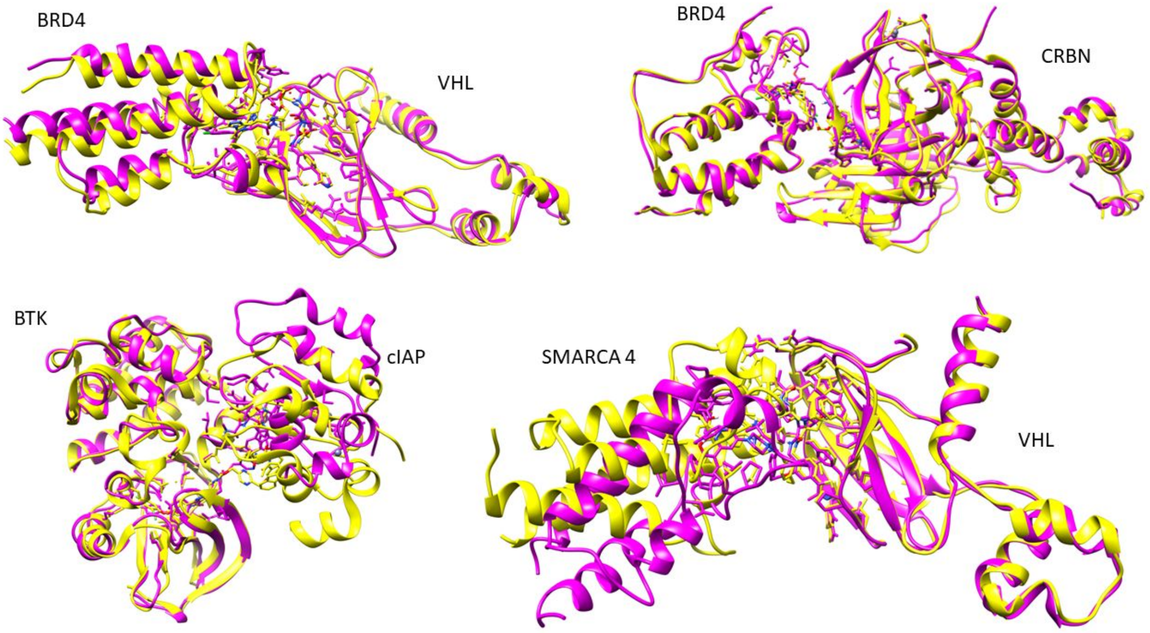

**Figure S3.** Protein-protein interactions of the TC models employed for the validation. A) Interactions of PROTAC dBET23 with BRD4 and CRBN in PDB 6BN7 and our model. B) Type and number of interactions in the crystal structures (PDB) and in the TC models obtained with DegraderTCM, C) Number of POI and E3L residues involved.

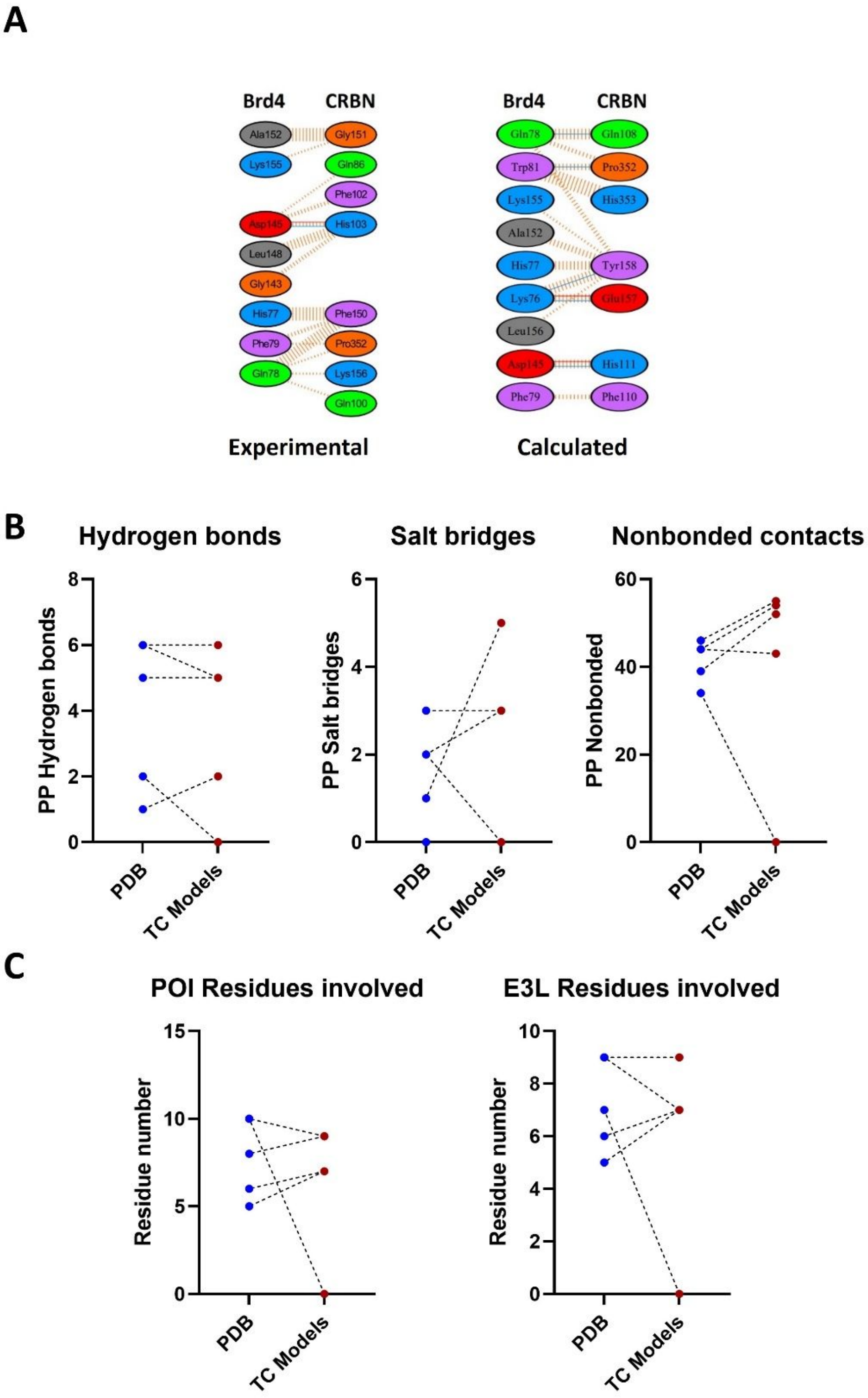
A
Brd4
CRBN
Ala152
Gly151
Lys155
Gln86
Phe102
Asp145
His103
Leu148
Gly143
His77
Phe150
Phe79
Pro352
Gln78
Lys156
Gln100
Experimental
Brd4
CRBN
Gln78
Gln108
Trp81
Pro352
Lys155
His353
Ala152
His77
Tyr158
Lys76
Glu157
Leu156
Asp145
His111
Phe79
Phe110
Calculated
B
Hydrogen bonds
PP Hydrogen bonds
Salt bridges
PP Salt bridges
Nonbonded contacts
PP Nonbonded
PDB
TC Models
C
POI Residues involved
E3L Residues involved
Residue number
PDB
TC Models

**Figure S4.** Summary of the interactions of the PROTAC MZ1 with Brd4, VHL and the PPIs between them. Ligand diagrams of PDB 5T35 and our modeled TC (A, B). PPI interactions from PDB 5T35 and our modeled TC (C, D).

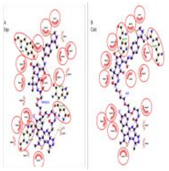

**Figure S5.** PPIs of PDB 5T35 (A) compared with our modeled TC of MZ1, Brd4, and VHL (B).

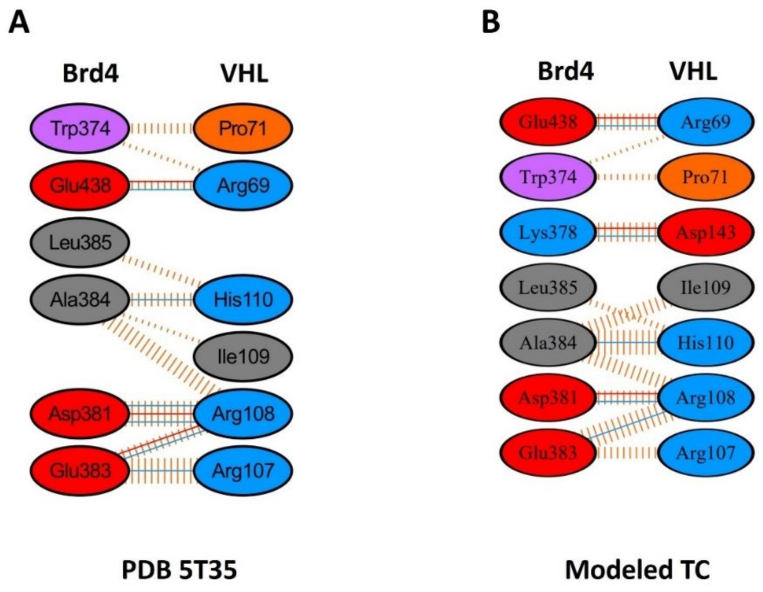

**Figure S6.** Summary of the interactions of the PROTAC macroPROTAC-1 with Brd4, VHL and the PPIs between them. Ligand diagrams of PDB 6SIS and our modeled TC (A, B). PPI interactions from PDB 6BN7 and our modeled TC (C, D).

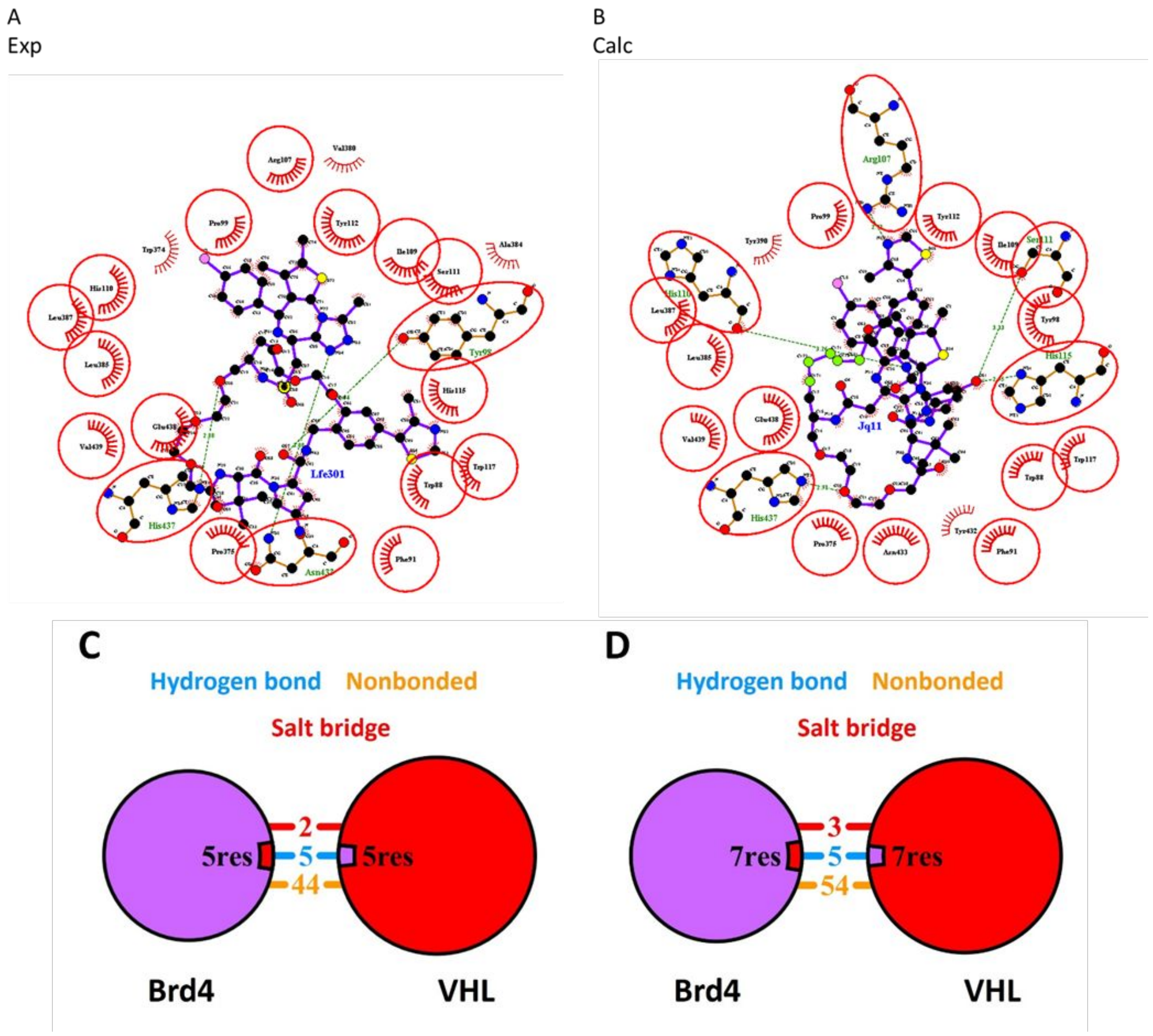

**Figure S7.** PPIs of PDB 6SIS (A) compared with our modeled TC of macroPROTAC-1, Brd4, and VHL (B).

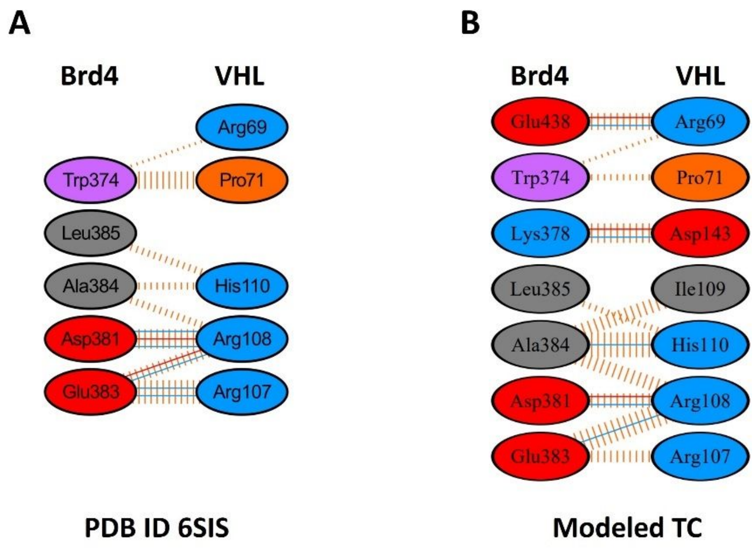

**Figure S8.** Summary of the interactions of the PROTAC PROTAC2 with SMARCA4, VHL and the PPIs between them. Ligand diagrams of PDB 6HR2 and our modeled TC (A, B). PPI interactions from PDB 6HR2. In our model PDBSum did not detect any contact.

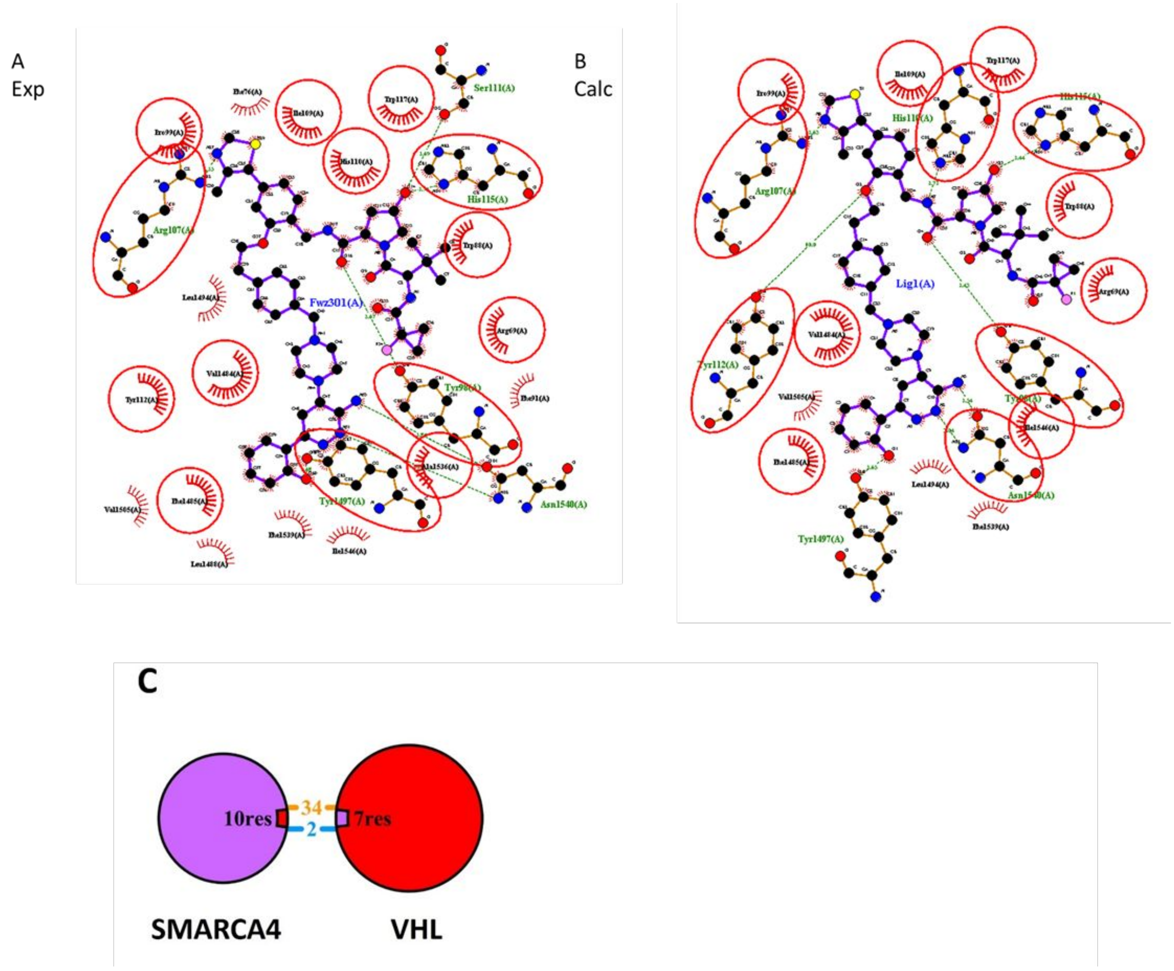

**Figure S9.** PPIs of PDB 6HR2. In our model PDBSum did not detect any contact.

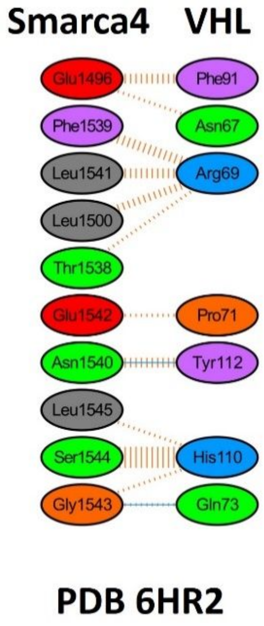

**Figure S10.** Summary of the interactions of the PROTAC compound-17 with BTK, cIAP and the PPIs between them. Ligand diagrams of PDB 6W7O and our modeled TC (A, B). PPI interactions from PDB 6W7O and our modeled TC (C, D),

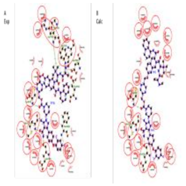

**Figure S11.** PPIs of PDB 6W7O (A) compared with our modeled TC of compound-17, BTK, cIAP (B).

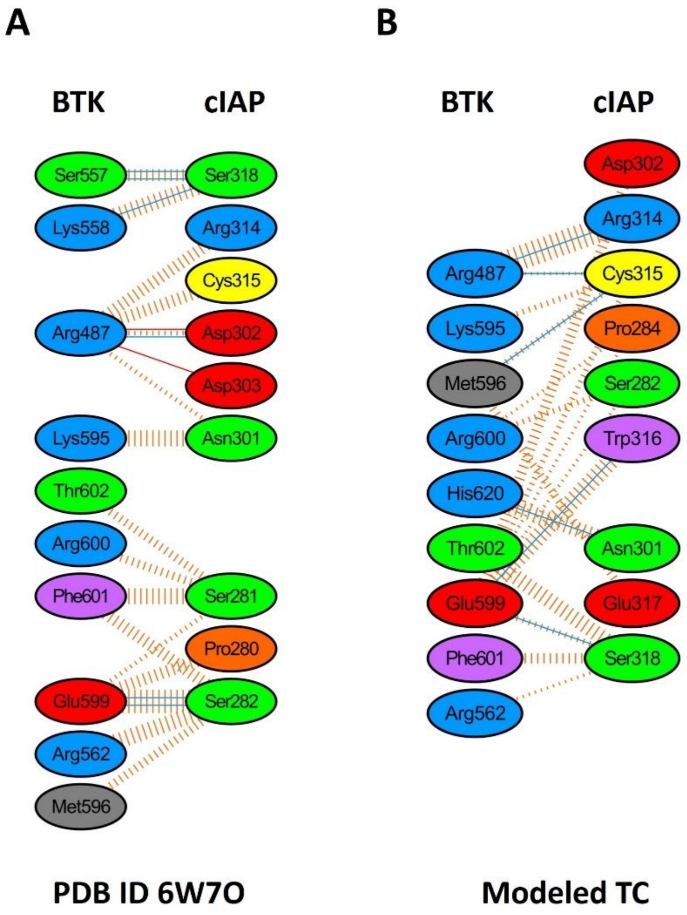

**Figure S12.** TC of XL-01126. A) Interactions of the warhead analogue, the inhibitor HG-10-102-01 (PDB ID 5OP4). B) interactions of the closest analog to the $L^{E3}$ (PDB ID 5NW2). C) whole PROTAC interactions. D) PPI interactions. Size of the spheres is proportional to the relative protein size; the size of the contact band is proportional to the interaction surface area.

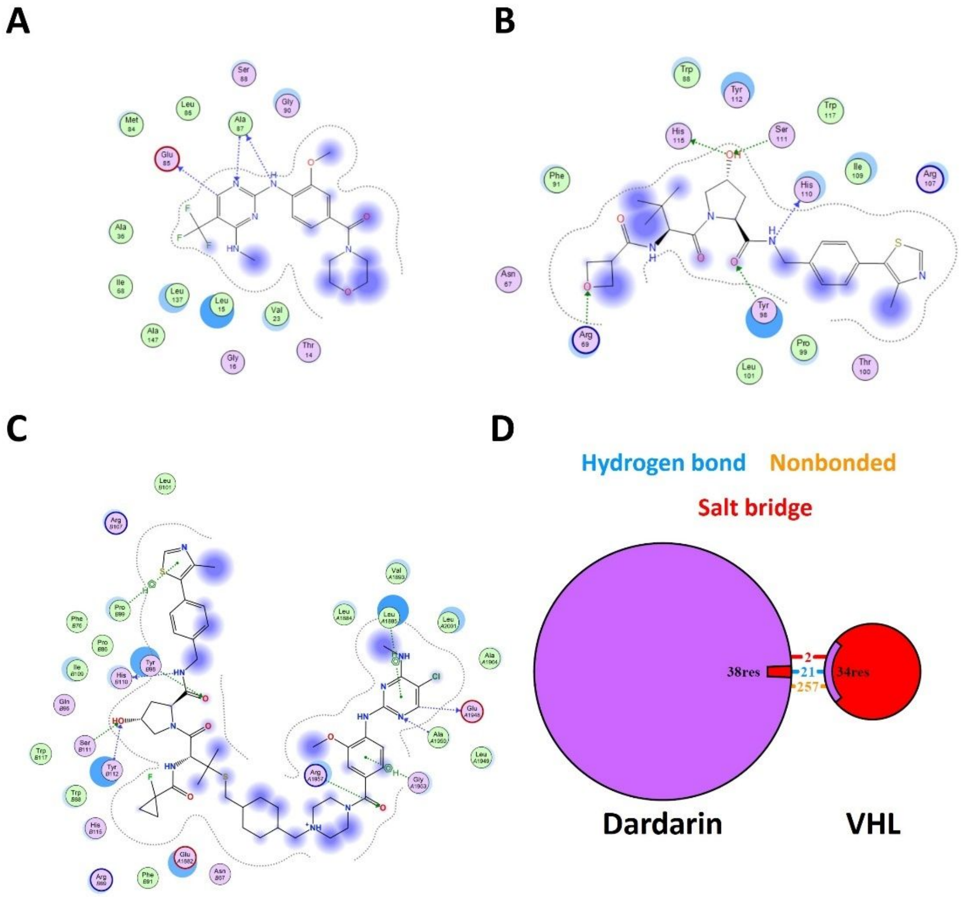

**Figure S13.** PPIs in the TC of XL-01126

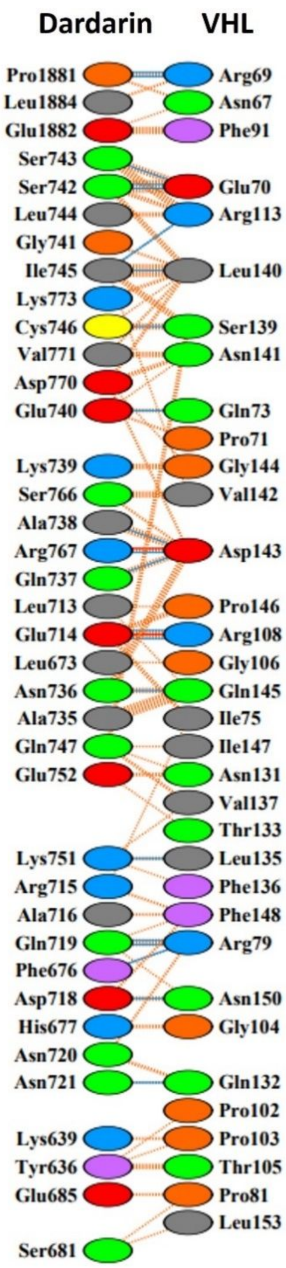

**Figure S14.** TC of XL-01168. A) Interactions of the warhead analogue, the inhibitor HG-10-102-01 (PDB ID 5OP4). B) interactions of the closest analog to the $L^{E3}$ (PDB ID 5NW2). C) whole PROTAC interactions. D) PPI interactions. Size of the spheres is proportional to the relative protein size; the size of the contact band is proportional to the interaction surface area.

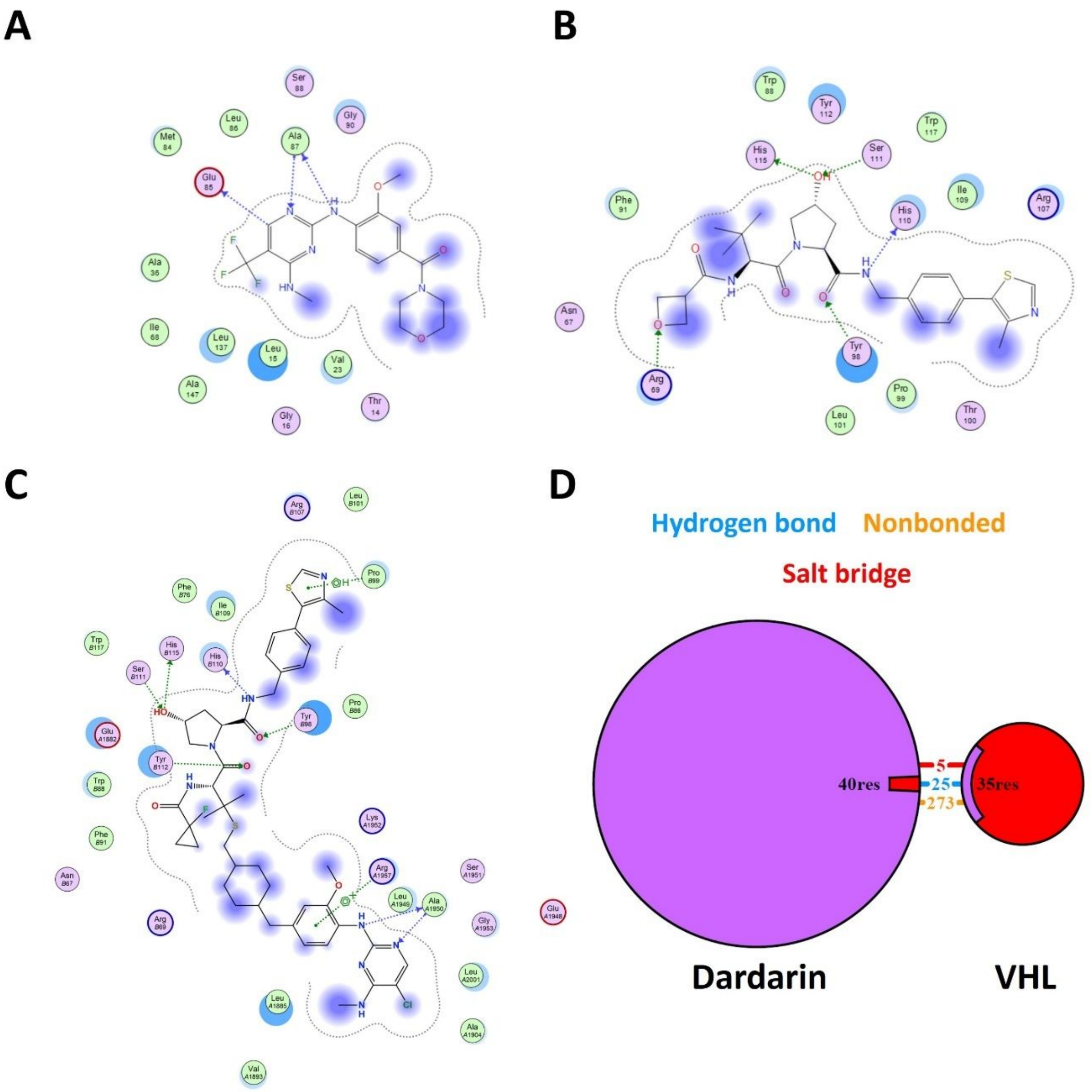

**Figure S15.** PPIs in the TC of XL-01168

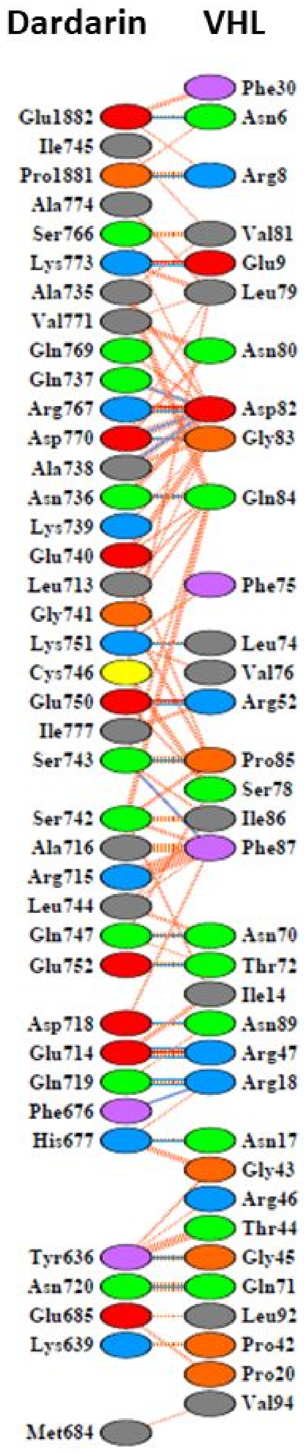
Dardarin
VHL
Phe30
Glu1882
Asn6
Ile745
Pro1881
Arg8
Ala774
Ser766
Val81
Lys773
Glu9
Ala735
Leu79
Val771
Gln769
Asn80
Gln737
Arg767
Asp82
Asp770
Gly83
Ala738
Asn736
Gln84
Lys739
Glu740
Leu713
Phe75
Gly741
Lys751
Leu74
Cys746
Val76
Glu750
Arg52
Ile777
Ser743
Pro85
Ser78
Ser742
Ile86
Ala716
Phe87
Arg715
Leu744
Gln747
Asn70
Glu752
Thr72
Ile14
Asp718
Asn89
Glu714
Arg47
Gln719
Arg18
Phe676
His677
Asn17
Gly43
Arg46
Thr44
Tyr636
Gly45
Asn720
Gln71
Glu685
Leu92
Lys639
Pro42
Pro20
Val94
Met684

**Figure S16.** TC of XL-01149. A) Interactions of the warhead analogue, the inhibitor HG-10-102-01 (PDB ID 5OP4). B) interactions of the closest analog to the $L^{E3}$ (PDB ID 5NW2). C) whole PROTAC interactions. D) PPI interactions. Size of the spheres is proportional to the relative protein size; the size of the contact band is proportional to the interaction surface area.

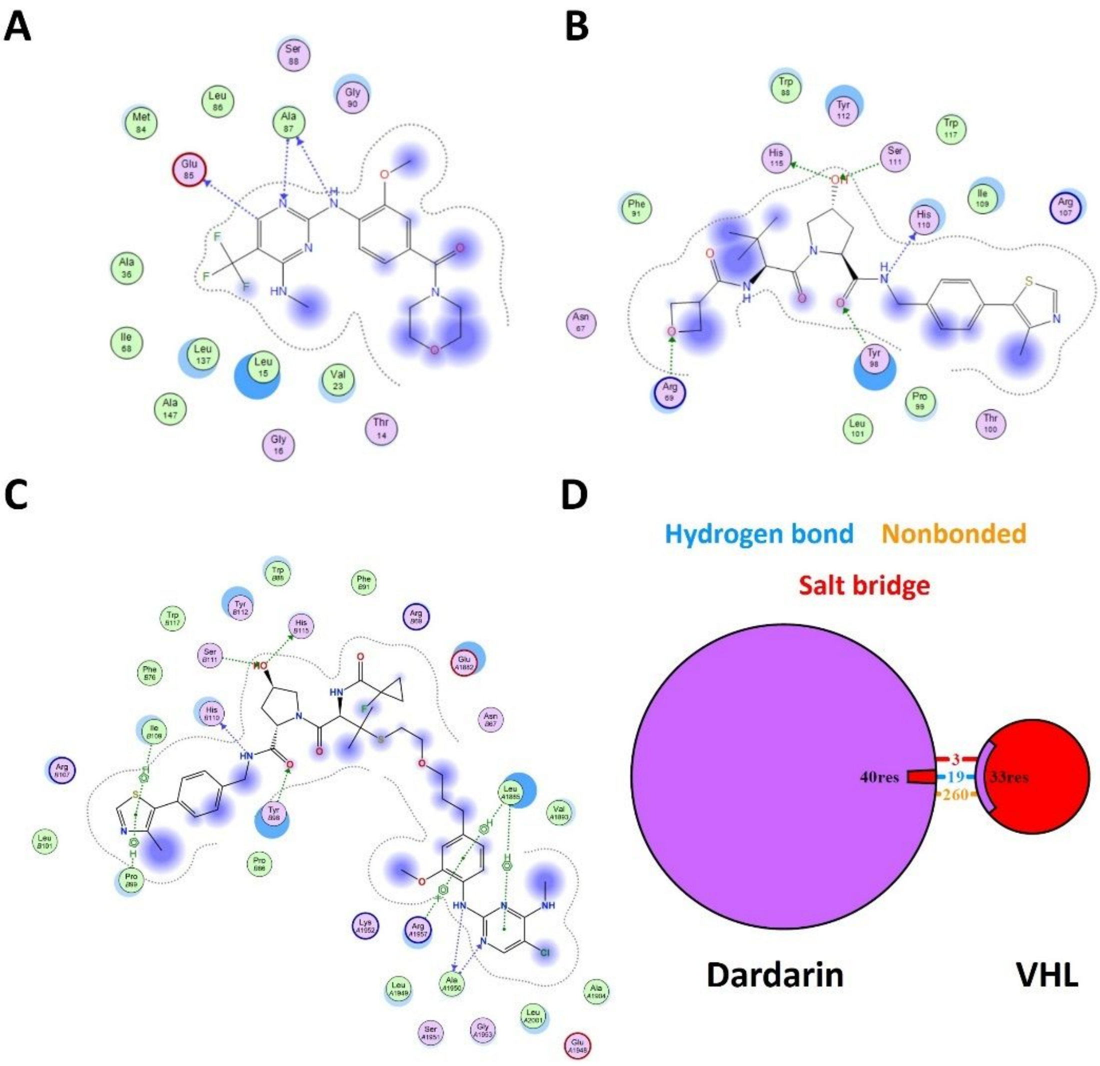

**Figure S17.** PPIs in the TC of XL-01149

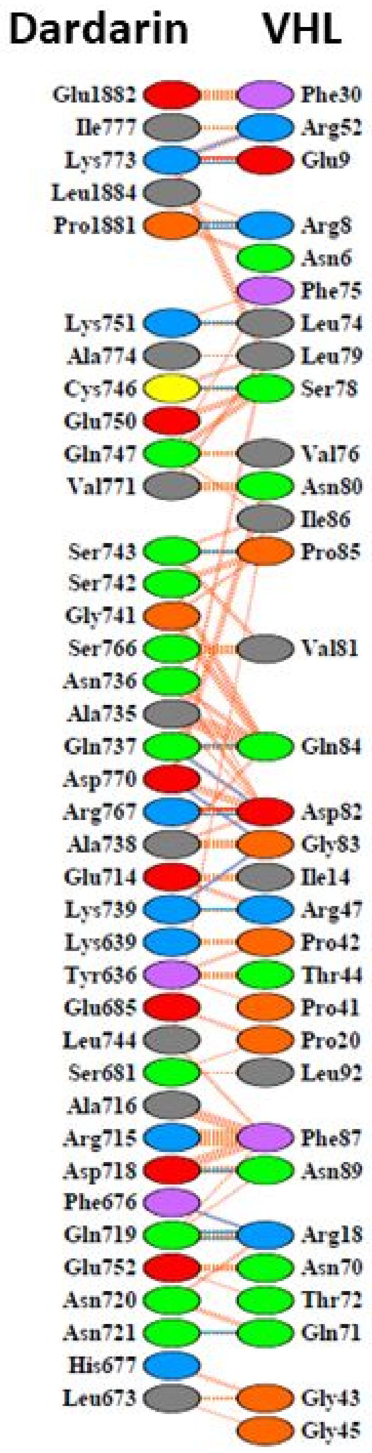

**Figure S18.** TC of XL-01076. A) Interactions of the warhead analogue, the inhibitor HG-10-102-01 (PDB ID 5OP4). B) interactions of the closest analog to the $L^{E3}$ (PDB ID 5NW2). C) whole PROTAC interactions. D) PPI interactions. Size of the spheres is proportional to the relative protein size; the size of the contact band is proportional to the interaction surface area.

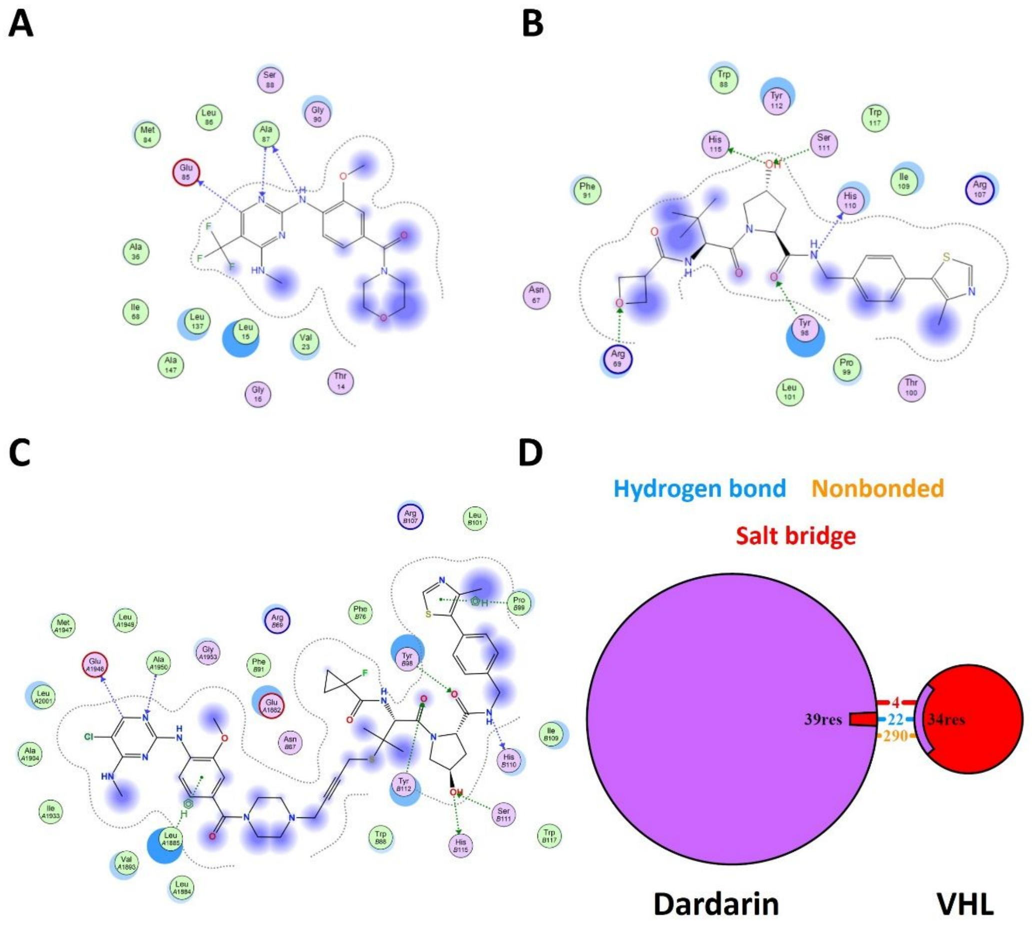

**Figure S19.** PPIs in the TC of XL-01076

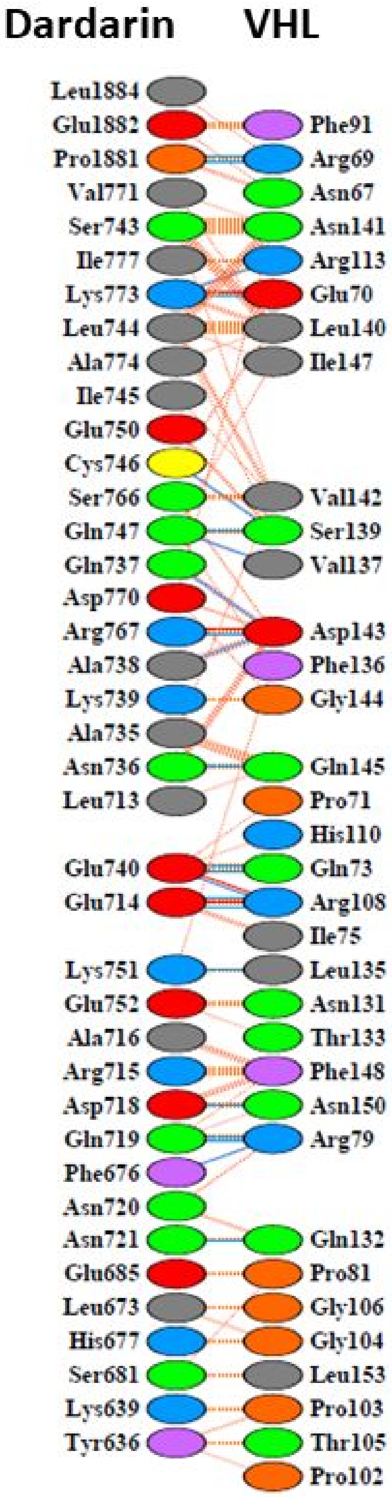
Dardarin
VHL
Leu1884
Glu1882
Pro1881
Val771
Ser743
Ile777
Lys773
Leu744
Ala774
Ile745
Glu750
Cys746
Ser766
Gln747
Gln737
Asp770
Arg767
Ala738
Lys739
Ala735
Asn736
Leu713
Glu740
Glu714
Lys751
Glu752
Ala716
Arg715
Asp718
Gln719
Phe676
Asn720
Asn721
Glu685
Leu673
His677
Ser681
Lys639
Tyr636
Phe91
Arg69
Asn67
Asn141
Arg113
Glu70
Leu140
Ile147
Val142
Ser139
Val137
Asp143
Phe136
Gly144
Gln145
Pro71
His110
Gln73
Arg108
Ile75
Leu135
Asn131
Thr133
Phe148
Asn150
Arg79
Gln132
Pro81
Gly106
Gly104
Leu153
Pro103
Thr105
Pro102

**Figure S20.** TC of XL-01118. A) Interactions of the warhead analogue, the inhibitor HG-10-102-01 (PDB ID 5OP4). B) interactions of the closest analog to the L$^{E3}$ (PDB ID 5NW2). C) whole PROTAC interactions. D) PPI interactions. Size of the spheres is proportional to the relative protein size; the size of the contact band is proportional to the interaction surface area.

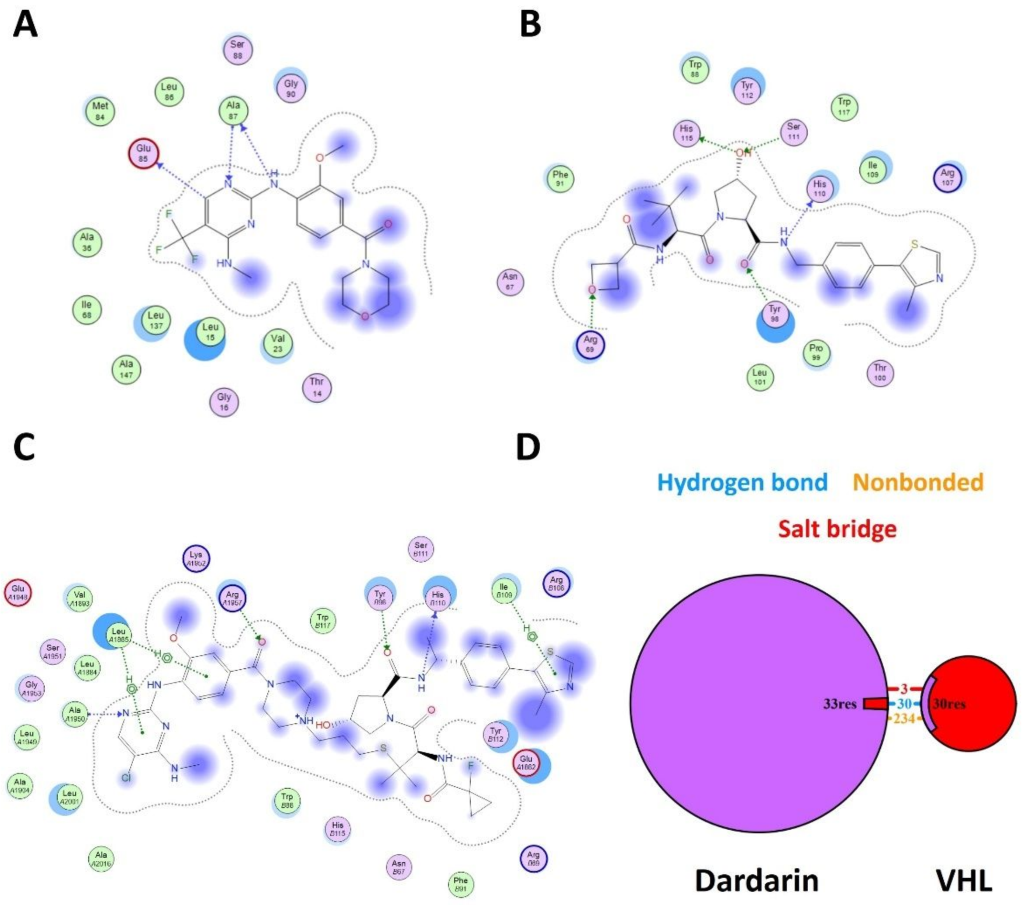

**Figure S21.** PPIs in the TC of XL-01118.

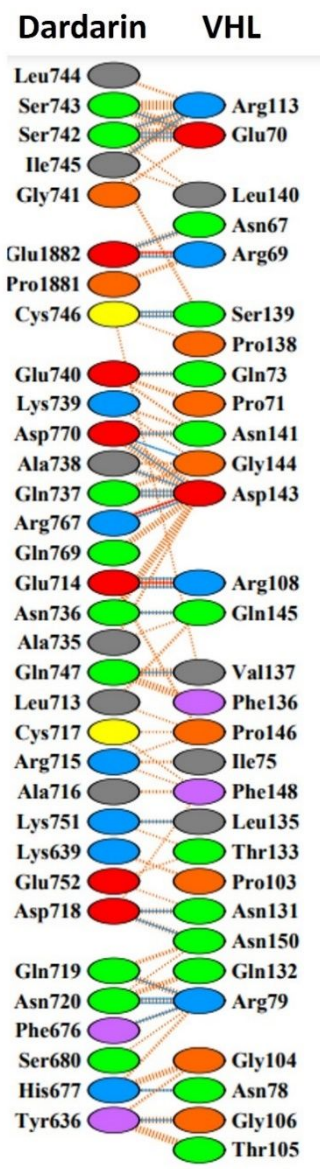

**Figure S22.** TC of ERD-308. A) Interactions of Raloxifene (closest analog to the warhead) in the X-Ray structure of ER (PDB ID 1ERR). B) interactions of the closest analog to the $L^{E3}$ from the co-crystal (PDB ID 5NW2). C) whole PROTAC interactions. D) PPI interactions. Size of the spheres is proportional to the relative protein size; the size of the contact band is proportional to the interaction surface area.

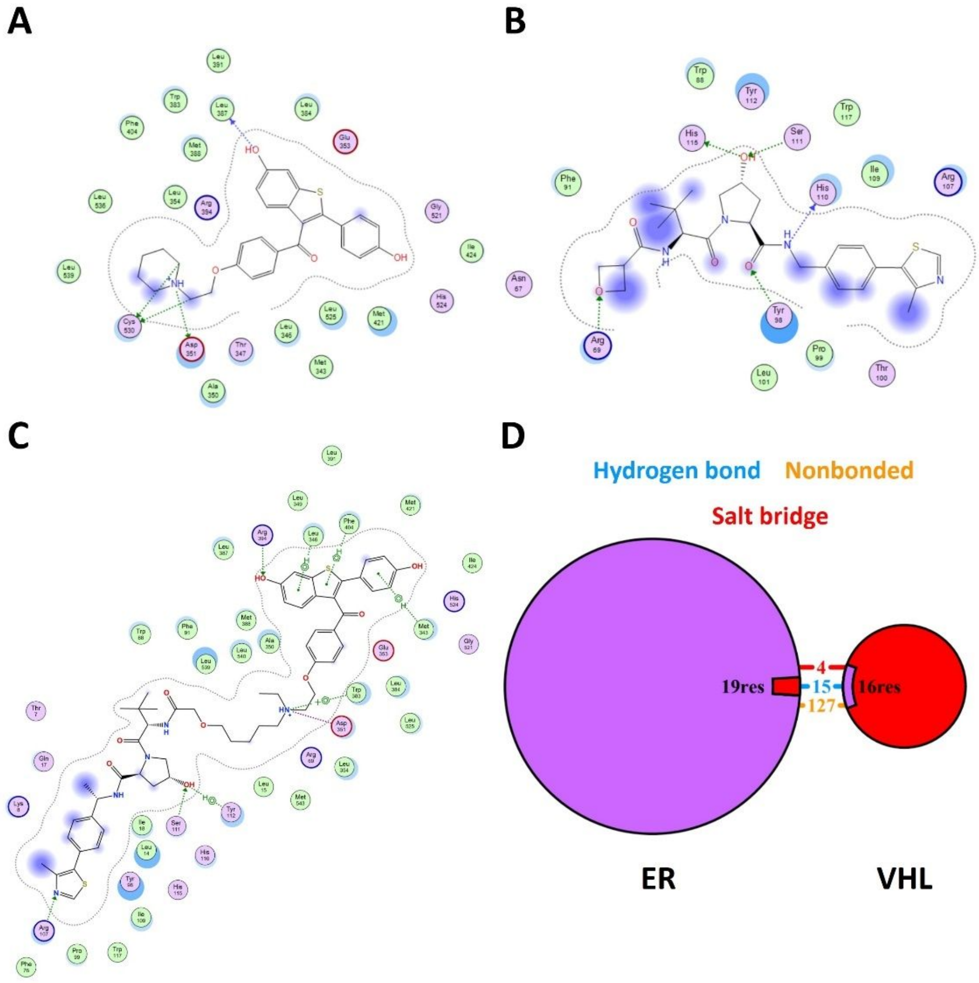

**Figure S23.** PPIs in the TC of ERD-308.

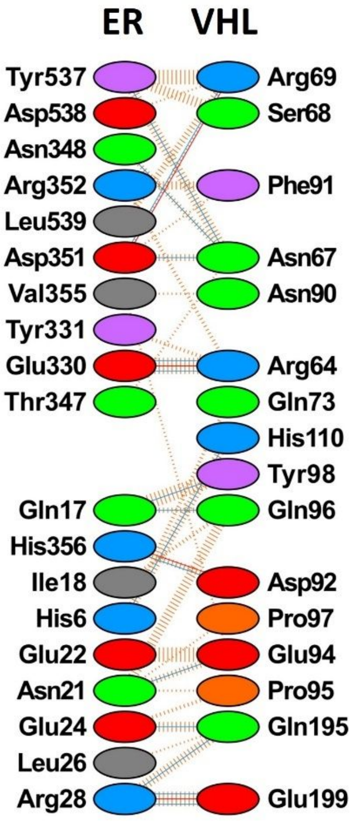

**Figure S24.** TC of ERD compound 17. A) Interactions of Raloxifene (closest analog to the warhead) in the X-Ray structure of ER (PDB ID 1ERR). B) interactions of the closest analog to the L$^{E3}$ from the co-crystal (PDB ID 5NW2). C) whole PROTAC interactions. D) PPI interactions. Size of the spheres is proportional to the relative protein size; the size of the contact band is proportional to the interaction surface area.

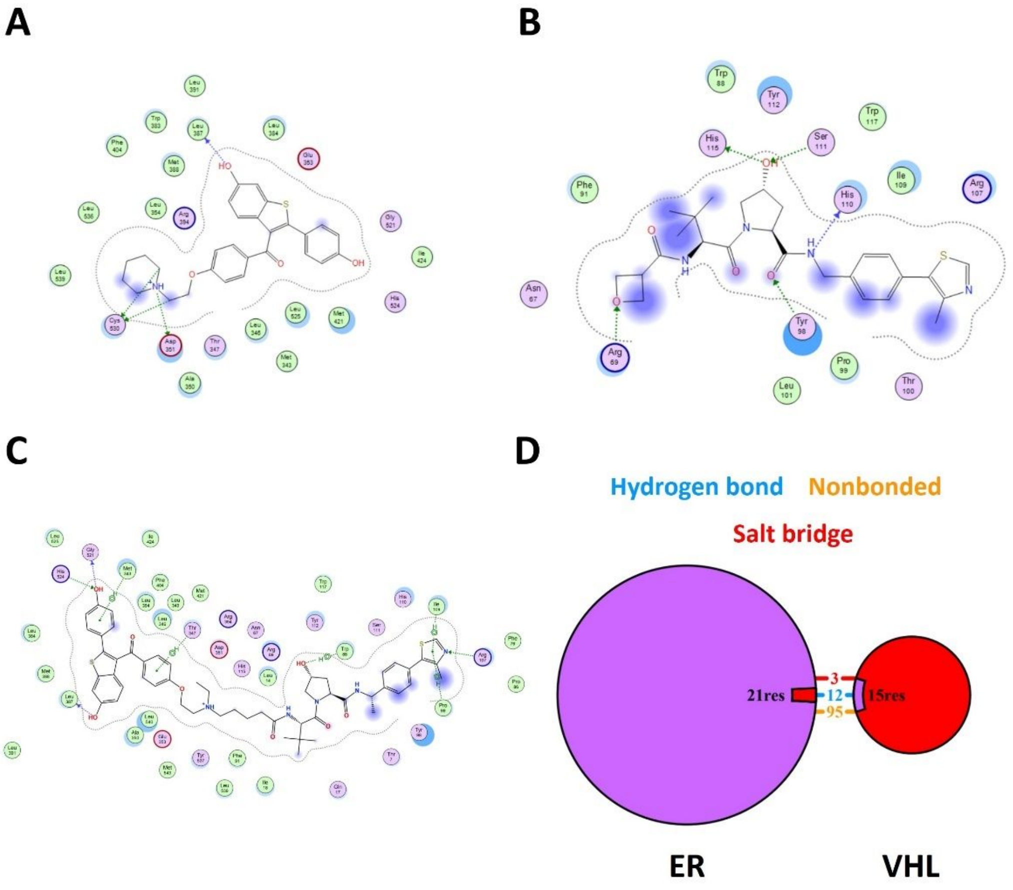

**Figure S25.** PPIs in the TC of ERD compound 17.

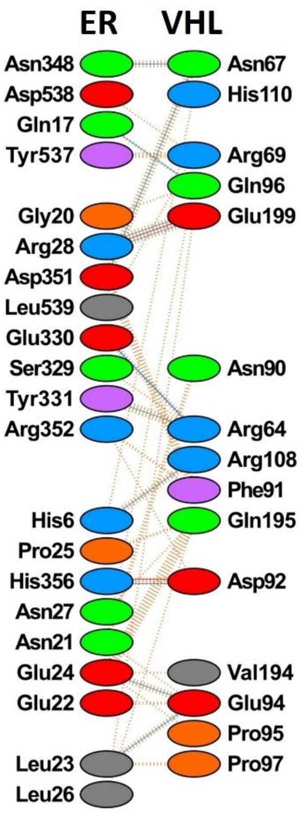

**Figure S26.** Interactions of A) ERD-C16 B) ERD-C18.

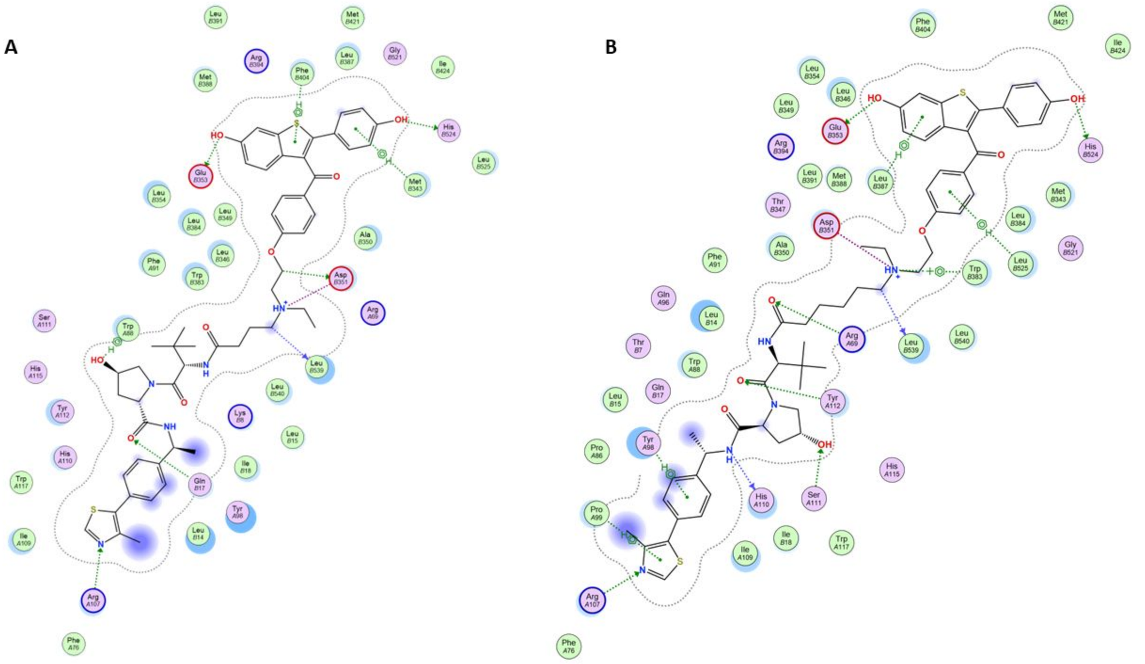

**Figure S27.** TC of ARV-110. A) Interactions of the warhead identified by docking in the X-Ray structure of AR (PDB ID 1Z95). B) interactions of the $L^{E3}$ from the co-crystal (PDB ID 4CI3). C) whole PROTAC interactions. D) PPI interactions. Size of the spheres is proportional to the relative protein size, the size of the contact band is proportional to the interaction surface area.

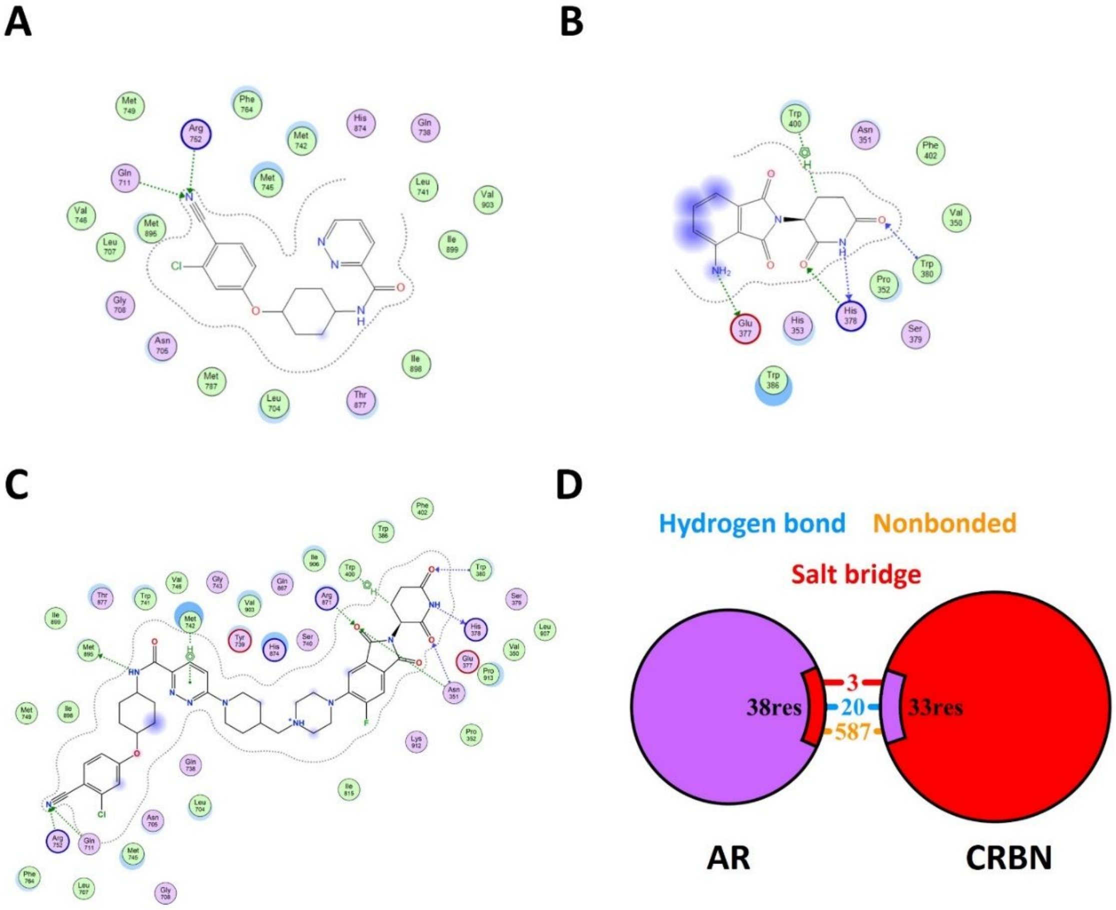

**Figure S28.** PPIs in the TC of ARV-110.

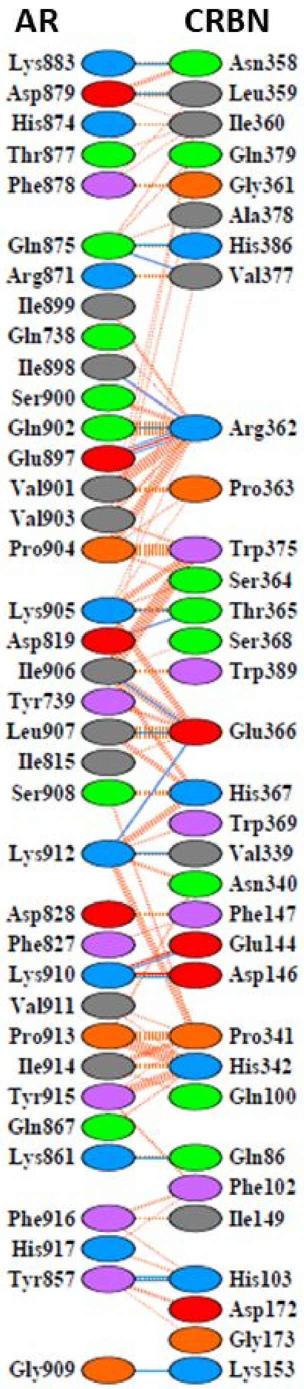

**Figure S29** TC of ARD-2585. A) Interactions of the warhead identified by docking in the X-Ray structure of AR (PDB ID 1Z95). B) interactions of the $L^{E3}$ from the co-crystal (PDB ID 4CI3). C) whole PROTAC interactions. D) PPI interactions. Size of the spheres is proportional to the relative protein size, the size of the contact band is proportional to the interaction surface area.

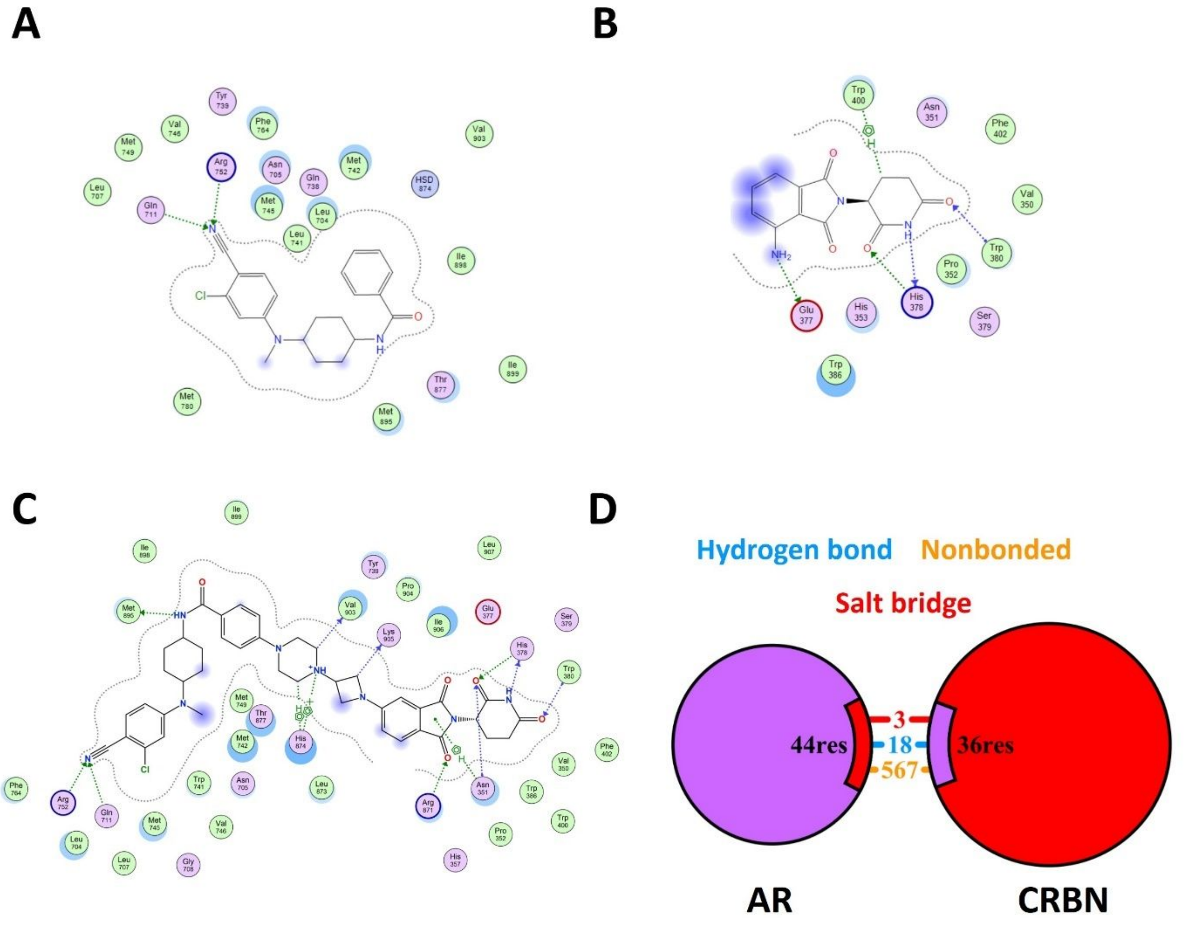

**Figure S30.** PPIs in the TC of ARD-2585.

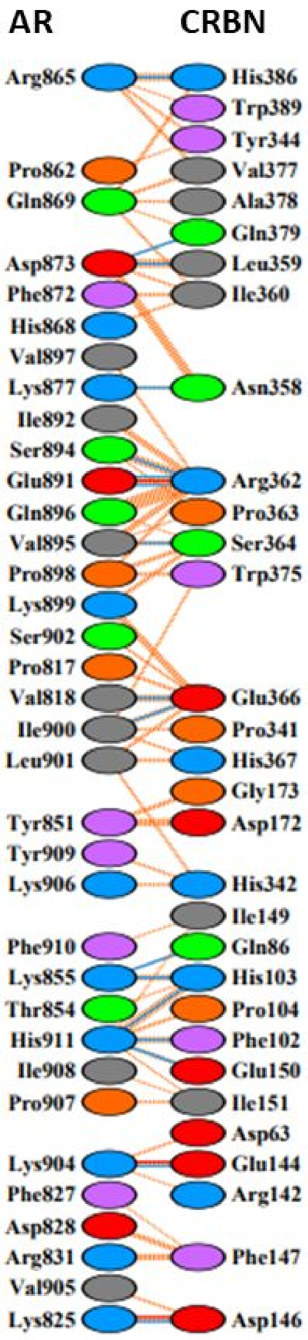

**Figure S31.** TC of AR-CRBN-33. A) Interactions of the warhead in the X-Ray structure of AR (PDB ID 1Z95). B) interactions of the $L^{E3}$. C) whole PROTAC interactions. D) PPI interactions. Size of the spheres is proportional to the relative protein size, the size of the contact band is proportional to the interaction surface area.

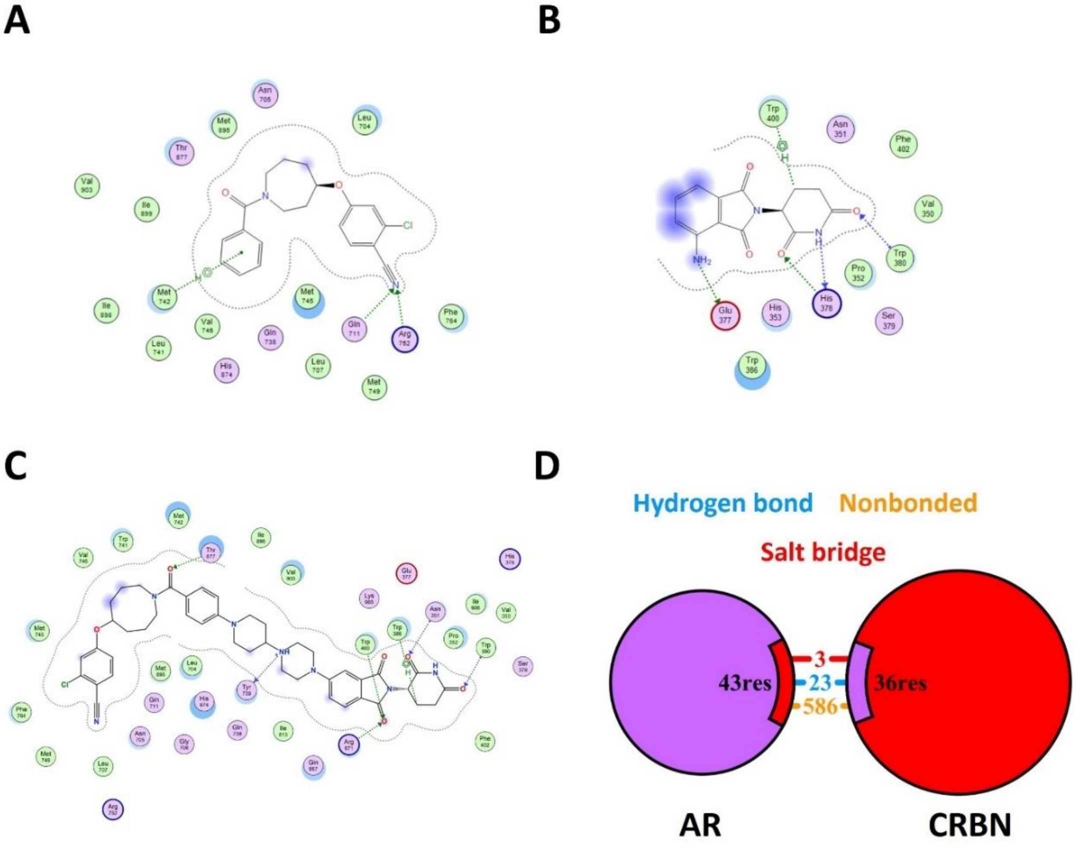

**Figure S32.** PPIs in the TC of AR-CRBN-33.

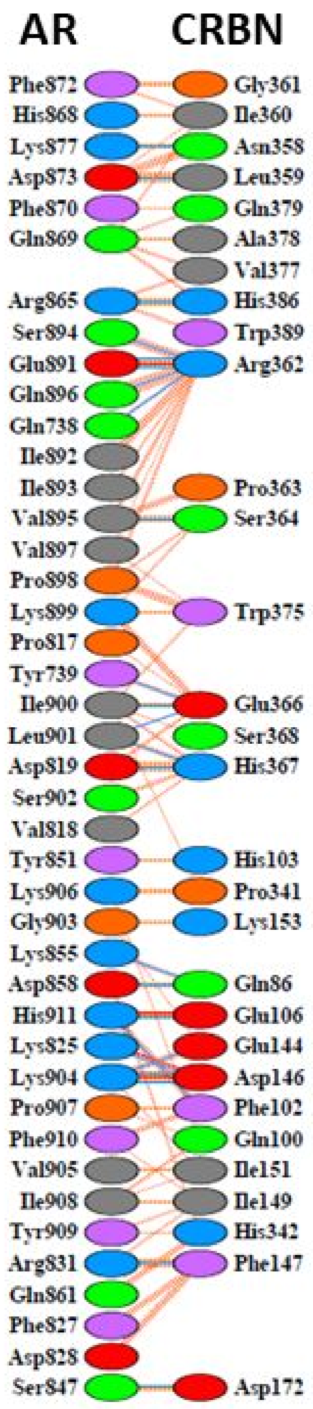

**Figure S33.** TC of ARD-266. A) Interactions of the warhead in the X-Ray structure of AR (PDB ID 1Z95). B) interactions of the closest analog to the $L^{E3}$ from the X-Ray structure (PDB ID 5NW2). C) whole PROTAC interactions. D) PPI interactions. Size of the spheres is proportional to the relative protein size, the size of the contact band is proportional to the interaction surface area.

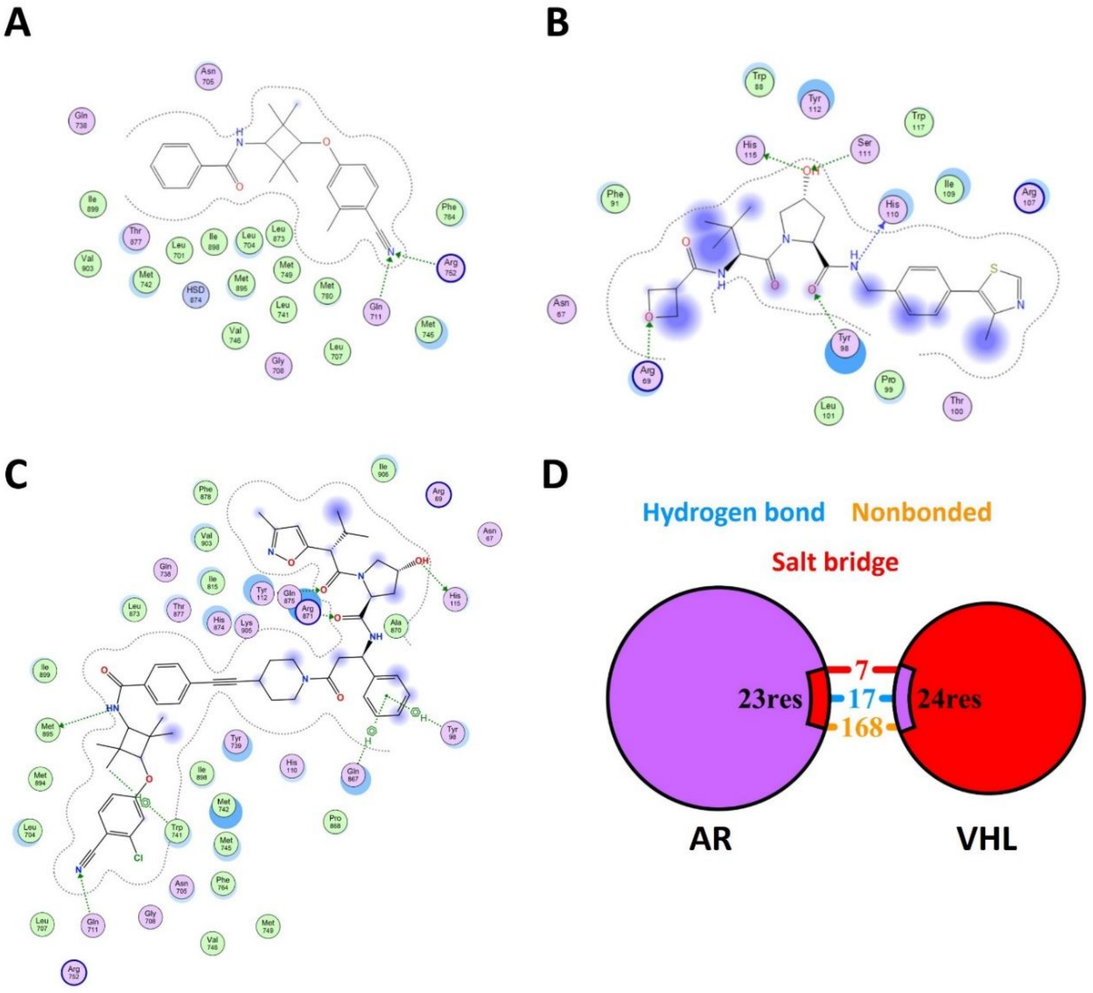

**Figure S34.** PPIs in the TC of ARD-266.

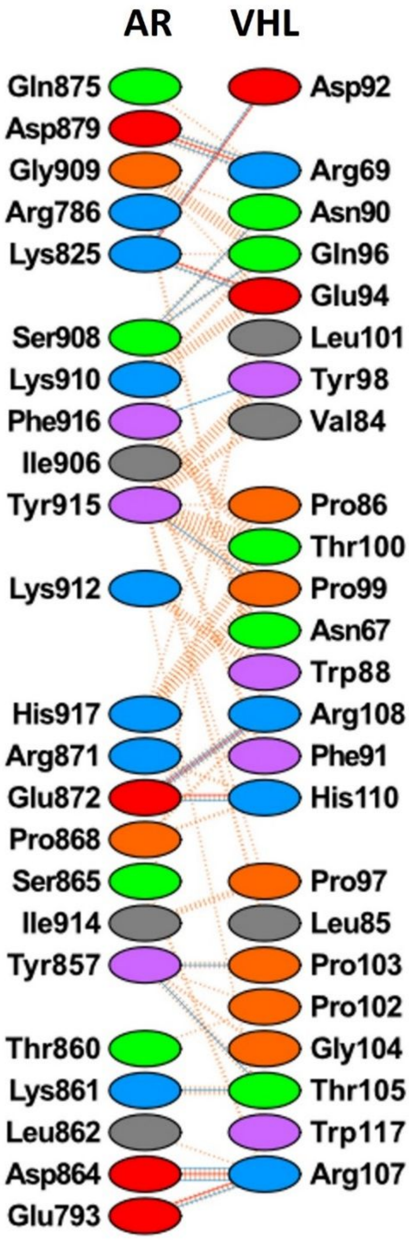

**Figure S35.** TC of AR-VHL-1-8. A) Interactions of the warhead in the X-Ray structure of AR (PDB ID 1Z95). B) interactions of the closest analog to the $L^{E3}$ from the X-Ray structure (PDB ID 5NW2). C) whole PROTAC interactions. D) PPI interactions. Size of the spheres is proportional to the relative protein size, the size of the contact band is proportional to the interaction surface area.

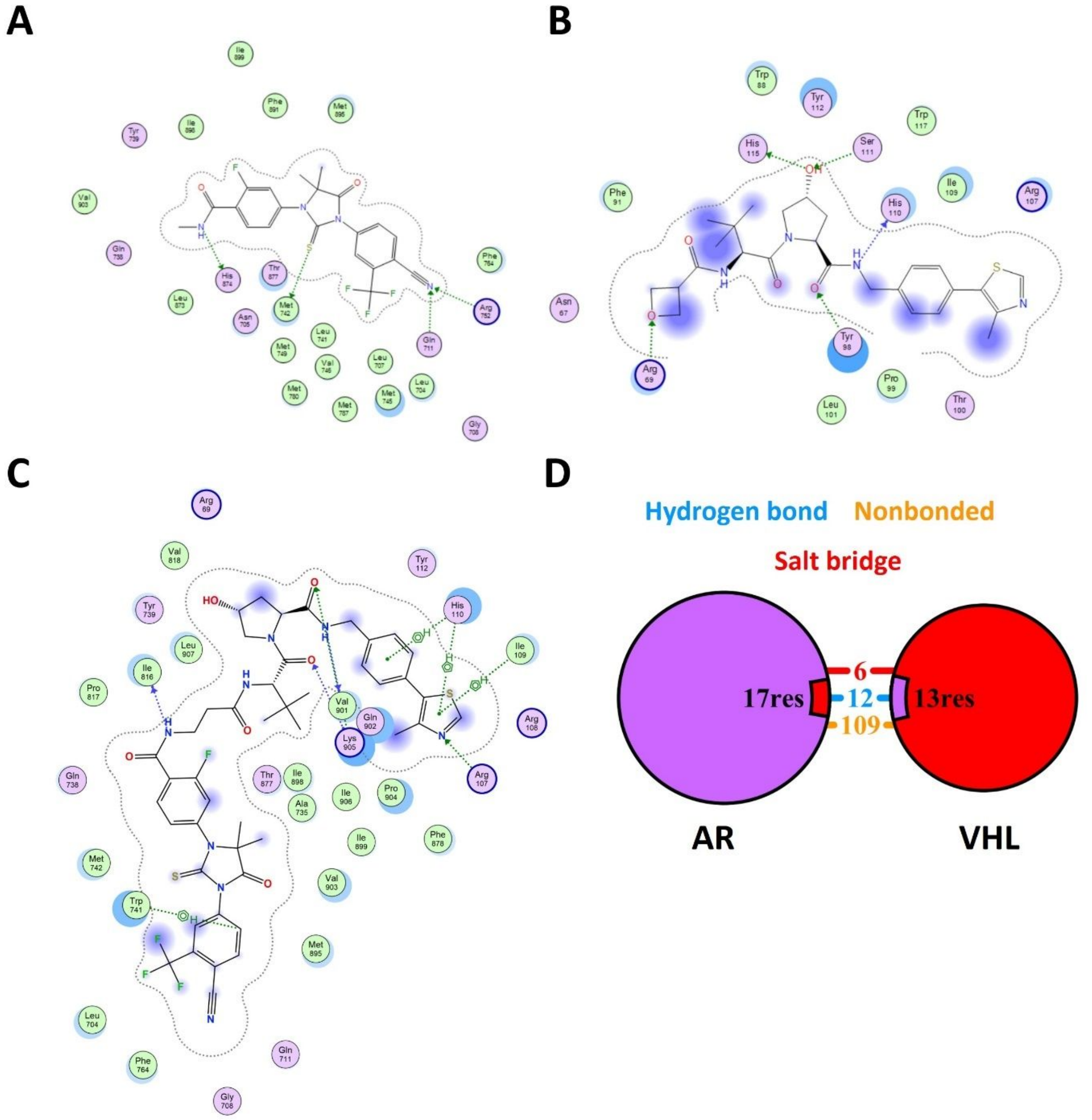
A
B
C
D
Hydrogen bond
Nonbonded
Salt bridge
6
12
109
17res
13res
AR
VHL

**Figure S36.** PPIs in the TC of AR-VHL-1-8.

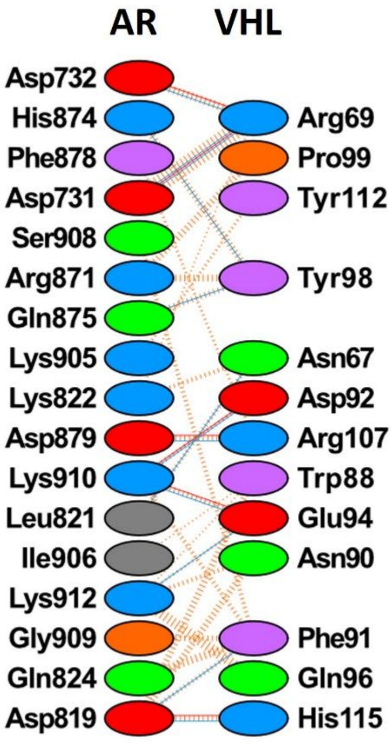

**Figure S37.** TC of ARV-471 based on ER AlphaFold model (UniProt IDs Q9UE35). A) Interactions of the warhead in the X-Ray structure of ER (PDB ID 2OUZ). B) interactions of the closest analog to the $L^{E3}$ from the co-crystal (PDB ID 4TZ4). C) whole PROTAC interactions. D) PPI interactions. Size of the spheres is proportional to the relative protein size; the size of the contact band is proportional to the interaction surface area.

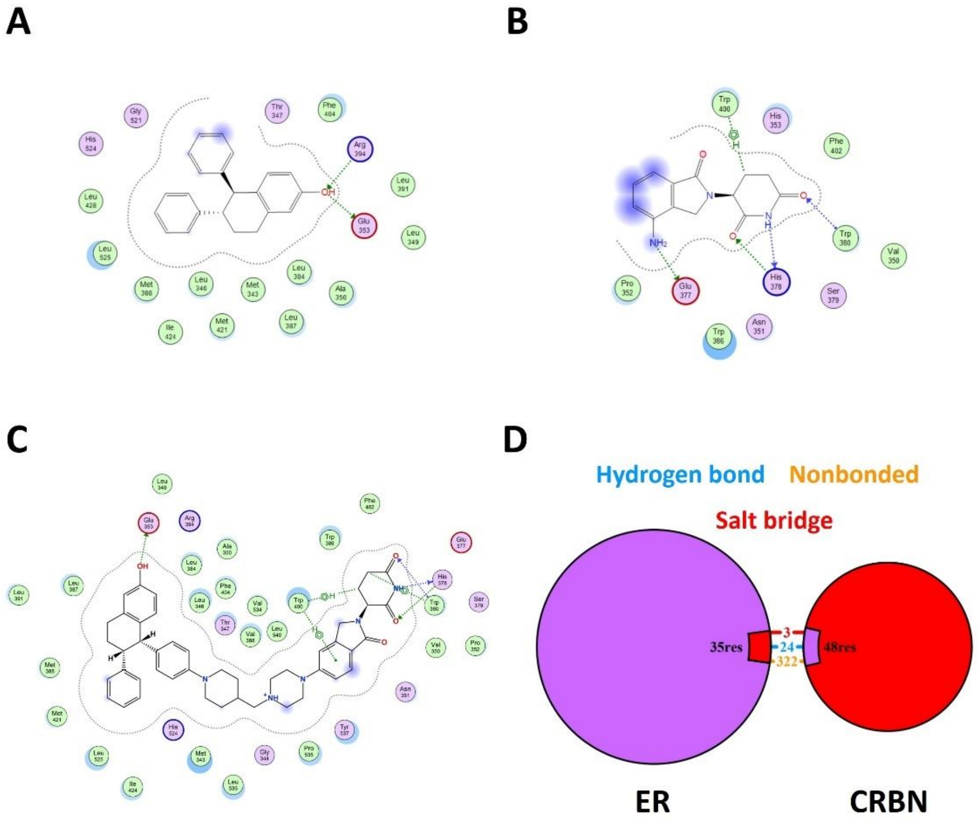

**Figure S38.** PPIs in the TC of ARV-471 based on ER AlphaFold model (UniProt IDs Q9UE35).

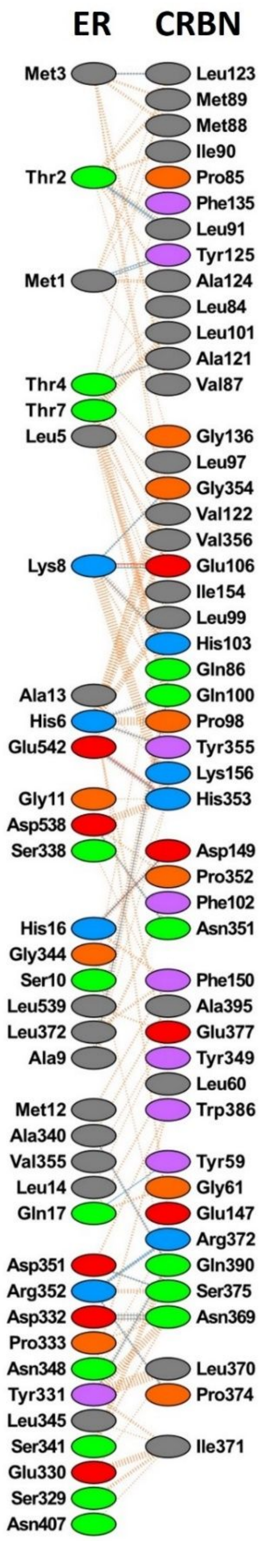

**Figure S39.** Number of PROTACs per target class with DC50 data. In blue are reported the discarded, while in orange the ones selected for further ternary complex modeling.

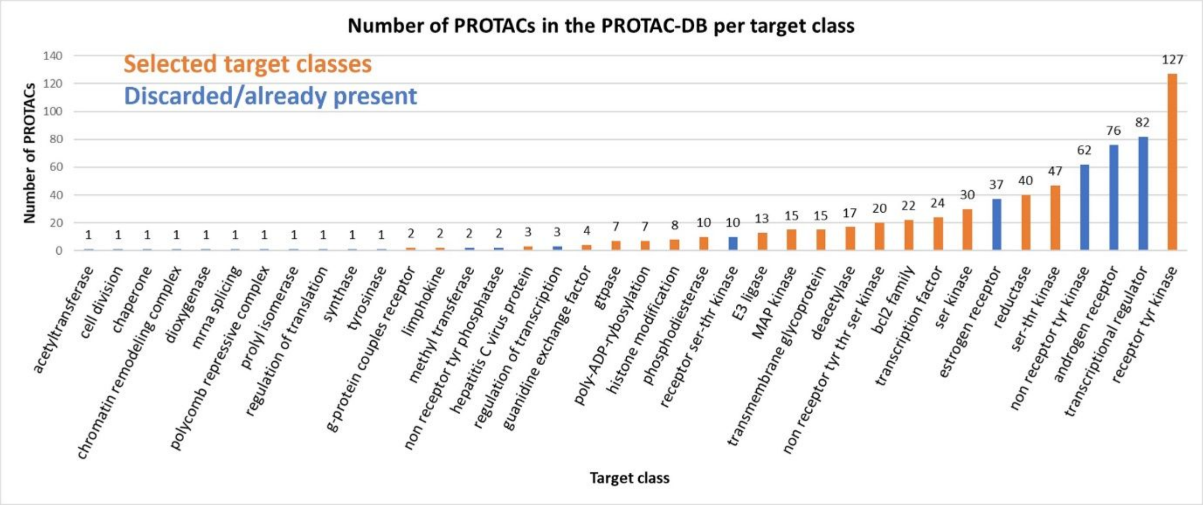

**Figure S40.** Target distribution among the classes. Each target chosen for the ternary complex modeling as representative for that class is highlighted in bold. The target chosen for ternary complex models are: ADRA1A (G-protein coupled receptor), SOS1 (GEF), PARP1 (poly-ADP-rybosylation), WDR5 (histone modification), PDEdelta (phosphodiesterase), CRBN (E3 ligase), p38delta (MAPK), BCL-xL (Bcl2 family), STAT3 (transcription factor), CDK4 (ser-kinase), HMGCR (reductase), EGFR (tyr-kinase receptor).

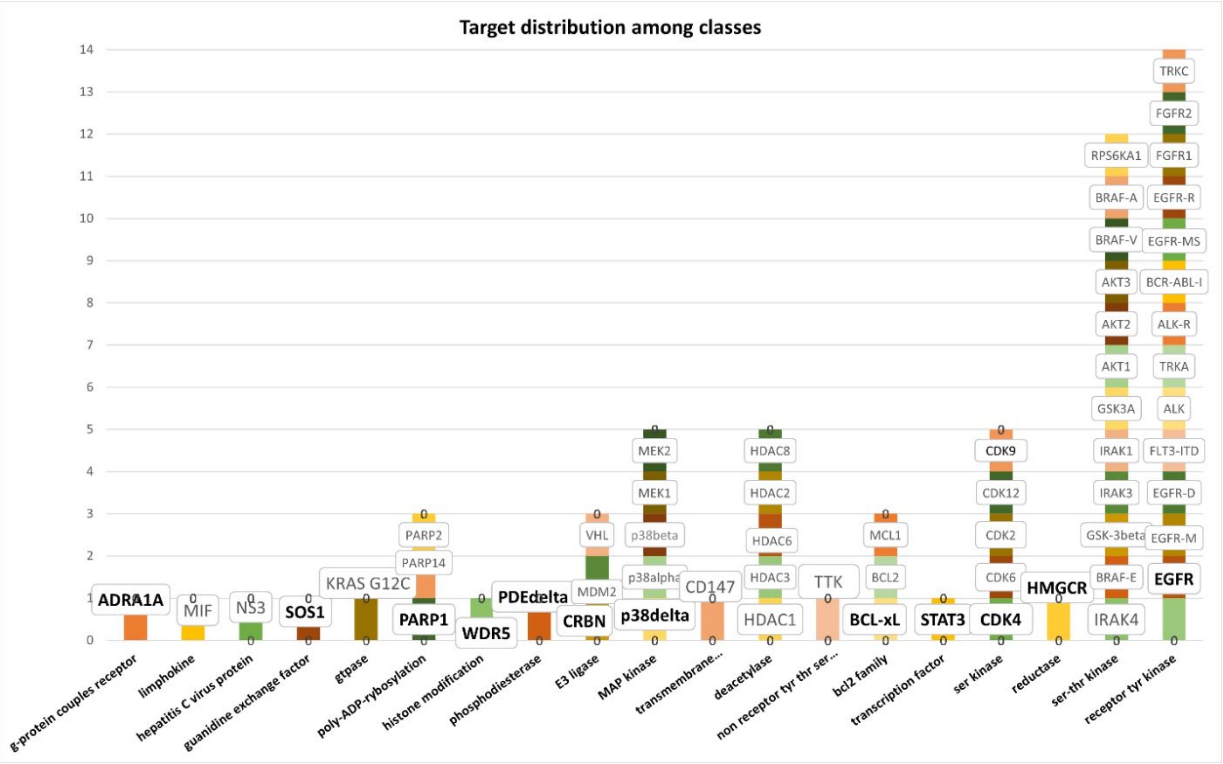

**Figure S41**. Venn diagram reporting interactions with residues of Lasofoxifene (Blue), Lenalidomide (Green), and ARV-471 (red). HB: Hydrogen Bond; NB: non-bonded contact.

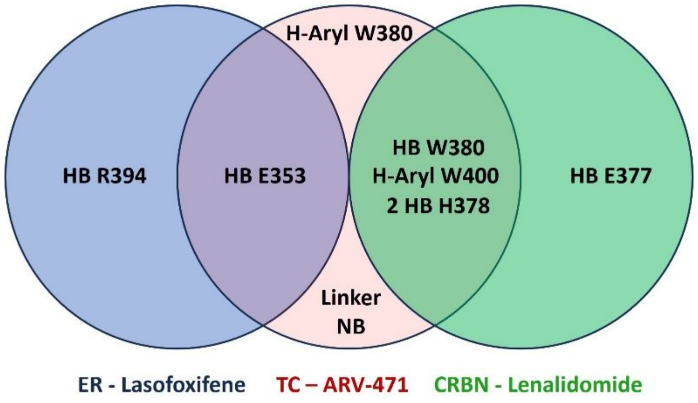


The TC of ARV-471, a CRBN-recruiting PROTAC of great interest, is the first one entering clinical trials. ARV-471 is composed by Lasofoxifene as warhead, a methylene bridged piperidine-piperazine linker, and Lenalidomide as CRBN binder (Fig S1). The structures of both molecules, co-crystallized with their protein targets are available (PDB codes 2OUZ and 4TZ4). In this case we sought for a more complete model and included the whole ER structure, as present in the AlphaFold database. The procedure involved all DegraderTCM steps followed by superposition of the AlphaFold model to the correspondent one in the TC. After unrestrained minimization, we observed no protein clashes, reinforcing the goodness of the TC and opening to the possibility of employing DegraderTCM to obtain more exhaustive TC models when coupled with AlphaFold predictions.

A thorough investigation of the interactions is beyond the scope of this work, but we can appreciate that the main ones in the co-crystals (Fig S37) are maintained in the ARV-471 TC model, supporting the stability of this TC, as summarized in the Venn diagram in Fig 7. We must mention that the two HBs differently recorded in TC and co-crystals display donor and acceptor groups still in proximity of the protein binding moieties (Fig S37C) and that the newly established interactions can be explained by the presence of the linker which results entirely hidden from the solvent, further supporting the TC stability. Stability is further corroborated by predicted interaction energies: the score, calculated on ARV-471 (-127 kcal/mol) is larger in module than the total ligand interaction energies of the corresponding co-crystal structures (-112 kcal/mol).

# Supplementary Tables

**Table S1.** PDB codes of reference structures.

| PDB code | Description |
|---|---|
| 6W7O | BTK-cIAP- compound 17 |
| 6SIS | Brd4-VHL-cPROTAC1 |
| 6HR2 | SMARCA-VHL-PROTAC 2 |
| 5T35 | Brd4-VHL-MZ1 |
| 5NW2 | VHL-VHL ligand 19 |
| 50P4 | GNE-7915-Dardarin (crystallographic surrogate of the kinase site) |
| 4TZ4 | CRBN- Lenalidomide |
| 4CI3 | DDB1-CRBN E3 ubiquitin ligase bound to Pomalidomide |
| 3ONI | BRD2 in complex with the inhibitor JQ1 |
| 3MXF | BRD4 in complex with the inhibitor JQ1 |
| 2OUZ | Lasofoxifene in complex with ER |
| 1Z95 | AR-Bicalutamide |
| 1ERR | ER in complex with Raloxifene |
| 6BN7 | Brd4-CRBN-dBet23 |
| 4QVX | BCL-xL warhead |
| 6d55 | SOS1 warhead |
| 4QL1 | WDR5 warhead |
| 3LQ8 | p38delta warhead |
| 5ML8 | PDEdelta warhead |
| 3U9Y | PARP1 warhead |
| 2ITO | EGFR warhead |
| 1HWK | HMGCR warhead |
| 2EUF | CDK6 warhead |
| 6NJS | STAT3 warhead |

**Table S2.** Degradation data from literature in case studies (Western Blot assays).

| compound | cell line | incubation time (h) | 1 nM WB bands (ER/GAPDH) | 33nM Degradation % | 1uM Degradation % | DC50 (nM) | Dmax% | Degradation capacity | DOI |
|---|---|---|---|---|---|---|---|---|---|
| XL01076 | WT MEFs | 24 | ND | ~20 | ~50 | ND | ND | poor degrader | https://doi.org/10.1021/jacs.2c05499 |
| XL01118 | WT MEFs | 24 | ND | ~30 | ~70 | ND | ND | poor degrader | https://doi.org/10.1021/jacs.2c05499 |
| XL01126 | WT MEFs | 24 | ND | ~65 | ~85 | ND | ND | strong degrader | https://doi.org/10.1021/jacs.2c05499 |
| XL01149 | WT MEFs | 24 | ND | ~10 | ~20 | ND | ND | poor degrader | https://doi.org/10.1021/jacs.2c05499 |
| XL01168 | WT MEFs | 24 | ND | ~0 | ~10 | ND | ND | poor degrader | https://doi.org/10.1021/jacs.2c05499 |
| ARV-110 | VCaP | 24 | ND | ND | ND | 1.6 | 98 | strong degrader | https://pubs.acs.org/doi/10.1021/acs.jmedchem.1c00900 |
| ARD-2585 | VCaP | 24 | ND | ND | ND | 0.1 | 98 | strong degrader | https://pubs.acs.org/doi/10.1021/acs.jmedchem.1c00900 |
| AR-CRBN 33 | VCaP | 24 | ND | ND | ND | >1000 | 22 | poor degrader | https://pubs.acs.org/doi/10.1021/acs.jmedchem.1c00900 |

| | | | | | | | | | |
|---|---|---|---|---|---|---|---|---|---|
| ARV-471 | MCF-7 | ND | ND | ND | ND | 1.8 | ND | strong degrader | https://www.arvinas.com/wp-content/uploads/2022/09/Hamilton-ASCO-2022.pdf |
| ERD-308 | MCF-7 | 4 | ~8 | ND | ND | 0.17 | 99 | strong degrader | https://doi.org/10.1021/acs.jmedchem.8b01572 |
| ER-C16 | MCF-7 | 4 | ~63 | ND | ND | ND | ND | poor degrader | https://doi.org/10.1021/acs.jmedchem.8b01572 |
| ER-C17 | MCF-7 | 4 | ~90 | ND | ND | ND | ND | poor degrader | https://doi.org/10.1021/acs.jmedchem.8b01572 |
| ER-C18 | MCF-7 | 4 | ~26 | ND | ND | ND | ND | strong degrader | https://doi.org/10.1021/acs.jmedchem.8b01572 |
| ER-C26 | MCF-7 | 4 | ~15 | ND | ND | ND | ND | strong degrader | https://doi.org/10.1021/acs.jmedchem.8b01572 |
| ARD-266 | LNCaP | 6 | ND | ND | ND | 0.5 | ND | strong degrader | https://doi.org/10.1021/acs.jmedchem.9b01393 |
| AR-VHL1-8 | LNCaP | 6 | ND | ND | 35 | ND | ND | poor degrader | https://doi.org/10.1021/acs.jmedchem.8b01631 |

**Table S3.** Additional PROTACs modeled and scored. In red the ones where DegraderTCM failed in predicting the degradation capacity.

| PROTAC DB ID | Target | Target class | E3 ligase | DC50 (nM) | Warhead energy | E3 energy | Total energy |
|---|---|---|---|---|---|---|---|
| 714 | PDEdelta | phosphodiesterase | CRBN | 48.00 | -101.3 | -40.1 | -141.40 |
| 1593 | PDEdelta | phosphodiesterase | CRBN | 30000.00 | -50.00 | -42.90 | -92.90 |
| 482 | STAT3 | transcription factor | CRBN | 50.00 | -409.00 | -60.00 | -469.00 |
| 495 | STAT3 | transcription factor | CRBN | 10000.00 | -354.90 | -58.10 | -413.00 |
| 1675 | PARP1 | poly-ADP-ribosylation | CRBN | 0.38 | -60.20 | -61.20 | -121.40 |
| 2101 | PARP1 | poly-ADP-ribosylation | CRBN | 6200.00 | -58.20 | -54.40 | -112.60 |
| 1108 | HMGCR | reductase | CRBN | 3000.00 | -120.70 | -30.50 | -151.20 |
| 1117 | HMGCR | reductase | CRBN | 100.00 | -125.00 | -33.20 | -158.20 |
| 176 | CRBN | E3 ligase | VHL | 1.50 | -52.10 | -79.40 | -131.50 |
| 1016 | CRBN | E3 ligase | VHL | 2546.10 | -35.90 | -54.10 | -90.00 |
| 742 | CDK6 | ser kinase | VHL | 200 | -48.8 | -94.7 | -143.50 |
| 2038 | CDK6 | ser kinase | VHL | 5.1 | -49.1 | -90 | -139.1 |
| 276 | EGFR | receptor tyr kinase | VHL | 11.70 | -27.00 | -83.40 | -110.40 |
| 576 | EGFR | receptor tyr kinase | VHL | 2000.00 | -67.90 | -39.20 | -107.10 |
| 181 | p38delta | MAP kinase | VHL | 51.40 | -61.80 | -88.50 | -150.30 |
| 187 | p38delta | MAP kinase | VHL | 1890.00 | -67.70 | -55.70 | -123.40 |
| 1840 | ADRA1A | g-protein coupled receptor | CRBN | 4320.00 | -32.70 | -17.10 | -49.80 |
| 1842 | ADRA1A | g-protein coupled receptor | CRBN | 2860.00 | -26.20 | -50.00 | -76.20 |
| 3245 | WDR5 | histone modification | VHL | 260.00 | -125.70 | -80.70 | -206.40 |
| 3255 | WDR5 | histone modification | VHL | 3.70 | -86.00 | -57.00 | -143.00 |
| 1649 | BCL-xL | bcl2 family | VHL | 4.80 | -138.30 | -88.10 | -226.40 |
| 3213 | BCL-xL | bcl2 family | VHL | 316.20 | -145.10 | -75.30 | -220.40 |

| | | | | | | | |
|---|---|---|---|---|---|---|---|
| **2628** | SOS1 | guanidine exchange factor | VHL | 216.40 | -187.40 | -82.10 | -269.50 |
| **2630** | SOS1 | guanidine exchange factor | VHL | 98.40 | -195.00 | -79.00 | -274.00 |
